# Deficiency of protein kinase Cδ reprograms macrophage immunometabolism to suppress pyroptosis and stabilize atherosclerotic plaques

**DOI:** 10.1038/s12276-026-01842-9

**Published:** 2026-09-10

**Authors:** Chih-Feng Lien, Hsin-Yi Chang, Shu-Han Yu, Rou-Ling Cho, Sy-Jou Chen, Tzu-Ting Kuo, Patrick Chun-Theng Chong, Chih-Hung Ye, Feng-Yen Lin, Wan-Lin Wu, Shih-Hua Lin, Chien-Sung Tsai, Chin-Sheng Lin

**Affiliations:** 1 Division of Cardiology, Department of Internal Medicine, Tri-Service General Hospital, National Defense Medical University, Taipei, Taiwan; 2 Graduate Institute of Medical Sciences, National Defense Medical University, Taipei, Taiwan; 3 Department of Research and Development, National Defense Medical University, Taipei, Taiwan; 4 Institute of Biotechnology, National Taiwan University, Taipei, Taiwan; 5 Department of Emergency Medicine, Tri-Service General Hospital, National Defense Medical University, Taipei, Taiwan; 6 Ph.D. Program for Cancer Molecular Biology and Drug Discovery, College of Medical Science and Technology, Taipei Medical University and Academia Sinica, Taipei, Taiwan; 7 Department of Internal Medicine, School of Medicine, Taipei Medical University, Taipei, Taiwan; 8 Division of Cardiology, Department of Internal Medicine, Taipei Medical University Hospital, Taipei, Taiwan; 9 Department of Discovery Science and Technology, Gilead Sciences, California, USA; 10 Division of Nephrology, Department of Internal Medicine, Tri-Service General Hospital, National Defense Medical University, Taipei, Taiwan; 11 Division of Cardiovascular Surgery, Department of Surgery, Tri-Service General Hospital, National Defense Medical University, Taipei, Taiwan

**Keywords:** Atherosclerosis, Mechanisms of disease, Energy metabolism

## Abstract

While atherosclerotic plaque vulnerability drives acute coronary syndrome, the regulatory mechanisms underlying plaque stability remain unclear. Protein kinase Cδ (PKCδ) has been implicated in atherosclerosis progression, but its specific role in plaque vulnerability and the underlying mechanisms require clarification. We analyzed PKCδ expression in human atheroma datas and investigated its role using low-density lipoprotein receptor-knockout mice with global and myeloid-specific PKCδ deletion. Plaque morphology, necrotic core formation, and fibrous cap thickness were evaluated. Macrophage metabolic profiling, mitochondrial function, inflammatory responses, and pyroptosis markers were assessed using biochemical and molecular approaches. Human atheroma analysis revealed elevated PKCδ expression, particularly in macrophages within ruptured plaques. PKCδ deletion in mice reduced necrotic core formation and increased fibrous cap thickness in both global and myeloid-specific models. Mechanistically, macrophage PKCδ deficiency improved mitochondrial fitness by promoting mitochondrial oxidative phosphorylation and elevating α-ketoglutarate (α-KG) levels. This metabolic shift reduced pro-inflammatory responses through PIK3AP1–mTORC2 activation and downregulated NLRP3 inflammasome-mediated pyroptosis. Dimethyl α-ketoglutarate (DKG) supplementation provided similar protective effects. Both human and in vivo analyses revealed PKCδ association with pyroptosis markers, including NLRP3, caspase-1, IL-1β, and GSDMD, whereas PKCδ deficiency reduced their co-localization with CD68⁺ macrophages. In conclusion, PKCδ serves as a key regulator of macrophage inflammation, pyroptosis, and mitochondrial dysfunction in atherosclerotic plaques. PKCδ deficiency promotes metabolic reprogramming that stabilizes plaques through reduced pyroptosis and enhanced mitochondrial function. These findings highlight PKCδ as a potential therapeutic target to reduce acute cardiovascular events by modulating plaque stability.

## Targeting PKCδ enhances plaque stability and mitochondrial health

Acute coronary syndrome is a major cause of death worldwide, primarily due to the rupture of vulnerable atherosclerotic plaques. This study explores the role of protein kinase Cδ (PKCδ) in macrophage-driven inflammation and plaque stability in atherosclerosis. Using in vitro and in vivo models, the researchers analyzed the effects of PKCδ deficiency on macrophage metabolism and inflammatory responses. They found that PKCδ deficiency reduces plaque size and complexity by enhancing oxidative phosphorylation and glycolysis, suppressing pro-inflammatory macrophage activity. This metabolic reprogramming, mediated through the PIK3AP1–mTORC2 pathway, decreases necrotic core formation and strengthens fibrous caps, suggesting PKCδ as a potential therapeutic target for stabilizing atherosclerotic plaques. These findings offer insights into the role of macrophage metabolism in plaque stability and highlight the potential of PKCδ in future cardiovascular therapies. "This summary was initially drafted using artificial intelligence, then revised and fact-checked by the author."

## Introduction

Acute coronary syndrome (ACS) remains a leading cause of mortality worldwide, and its pathogenesis is attributed primarily to the rupture of vulnerable atherosclerotic plaques^1^. Inflammation is recognized as a pivotal contributor to the initiation, progression, and destabilization of these plaques, playing a central role in the pathophysiology of cardiovascular disease. The clinical relevance of inflammation is further supported by accumulating evidence that links inflammatory activity to adverse cardiovascular outcomes, as demonstrated by the CANTOS (Canakinumab Anti-inflammatory Thrombosis Outcomes Study) trial and subsequent colchicine-based clinical trials^2–4^. Accordingly, comprehensive analyses of inflammation-driven molecular mechanisms and the development of targeted therapies are crucial for advancing ACS management.

Macrophages contribute to atherosclerotic plaque evolution through key inflammatory processes^5^. Single-cell RNA sequencing (scRNA-seq) has identified multiple macrophage subpopulations within atherosclerotic plaques, including metabolically dysfunctional foamy macrophages in human lesions and lipid-metabolic or -catabolic macrophages in murine plaques^6,7^. Metabolic reprogramming serves as a fundamental determinant of macrophage polarization and subsequently drives the pathophysiology of atherosclerosis. The metabolic shift toward enhanced glycolysis, coupled with impaired tricarboxylic acid (TCA) cycle activity and oxidative phosphorylation (OXPHOS), orchestrates M1-like polarization and triggers pro-inflammatory activation in macrophages^8^. Conversely, metabolic profiles favoring OXPHOS activity promote M2 polarization^9^. The metabolically programmed M1 macrophages perpetuate plaque instability through matrix metalloproteinase secretion, extracellular matrix degradation, and subsequent programmed cell death, ultimately contributing to the development of thin fibrous cap and necrotic core^10^. These insights highlight that therapeutic strategies specifically targeting the metabolic pathways governing macrophage polarization represent a promising approach for controlling vascular inflammation and achieving plaque stabilization.

Protein kinase Cδ (PKCδ) is a member of the protein kinase C (PKC) family, which consists of serine/threonine kinases involved in various cellular processes, including differentiation, proliferation, inflammation, and apoptosis^11^. Our previous studies have shown that PKCδ regulates the expression of the macrophage scavenger receptors SR-A and CD36, thereby increasing the uptake of oxidized low-density lipoprotein and exacerbating foam cell formation^12^. In vivo experiments have indicated that PKCδ deficiency enhances vein graft atherosclerosis in mice^13^, while the absence of macrophage PKCδ increases atherogenic diet-induced atherosclerosis in *Apoe*^*-*^^*/-*^ mice^14^. In a human study, compared with normal arteries, the atherosclerotic left internal mammary artery exhibited a significant increase in PKCδ protein expression^12^. These results indicate that targeting PKCδ may help stabilize atherosclerotic plaques, illustrating the complex and distinct roles of PKCδ in various cell types involved in atherosclerosis, a multifaceted process that remains incompletely understood.

This study investigated the role of PKCδ in M1 macrophage-mediated inflammatory responses and its impact on plaque stability in atherosclerosis. Using in vitro and in vivo models, metabolite analysis, human data analysis, and advanced omics technologies, including transcriptomics and phosphoproteomics, we aimed to validate the effects of macrophage PKCδ on atherosclerotic plaque evolution. Our findings reveal that macrophage PKCδ regulates inflammatory responses, metabolic reprogramming, and cell death, thereby highlighting its therapeutic potential in atherosclerosis.

## Methods

An extended description of methods is provided in the Supplementary material.

### Animals and in vivo experiments

PKCδ-deficient (*PKCδ*^*-/-*^) mice were obtained from Professor Keiichi I. Nakayama^15^ and crossbred with low-density lipoprotein receptor-deficient (*Ldlr*^*-/-*^) mice (RRID:IMSR_JAX:002207) to generate *Ldlr*^*-/-*^+*PKCδ*^*-/-*^ mice. C57BL/6J wild-type (WT; RRID:IMSR_JAX:000664) mice were obtained from the National Laboratory Animal Center, Taiwan. All animals were bred and maintained under pathogen-free conditions in accordance with a protocol approved by the Laboratory Animal Center at the National Defense Medical University (Protocol No. IACUC-20072). All experimental procedures complied with the NIH Guide for the Care and Use of Laboratory Animals. Every effort was made to minimize animal suffering and to use the minimum number of animals required to achieve scientific objectives. A high-cholesterol diet (HCD) comprising 34.1% sucrose, 20% milk fat, and 0.3% cholesterol (D02031507; Research Diets, Inc.) was used in this study. Eight-week-old mice were fed HCD for 12 weeks. At 20 weeks of age, the mice were anesthetized with tiletamine–zolazepam (20 mg/kg) and xylazine (5 mg/kg) injected intraperitoneally before blood collection via cardiac puncture. After perfusion with 20 ml of phosphate-buffered saline, the liver was harvested for quantitative polymerase chain reaction and western blot analysis, and the aorta, heart, and liver were fixed in 4% paraformaldehyde for histology and immunohistochemistry (IHC) staining. Blood samples were centrifuged at 3,000×*g* for 15 minutes at 4 °C, and the plasma was collected and stored at −80 °C.

### Bone marrow transplantation

Bone marrow (BM) cells were isolated from 8–12-week-old WT and *PKCδ*^*-/-*^ mice, after which 2 × 10^6^ cells/100 μl were intravenously injected into lethally irradiated (2 × 500 rad) *Ldlr*^*-/-*^ recipient mice. Following bone marrow transplantation (BMT), the mice received antibiotics in their drinking water and were allowed a 4-week recovery period to facilitate BM reconstitution. After 4 weeks, blood was collected from the recipients via the submandibular (facial) vein for genotyping and verification of BM reconstitution. For atherosclerosis experiments, all recipient mice were fed HCD for 12 weeks.

### Statistical analysis

All experiments were performed with at least three independent replicates. Data are presented as the mean ± s.e.m., as indicated in each graph. Statistical analyses were conducted using GraphPad Prism 9.0 (GraphPad Software). Comparisons between multiple groups were performed using a two-way analysis of variance with Tukey’s post hoc test. Comparisons between two groups were performed using two-tailed Student’s *t* tests. *A P* value < 0.05 was considered statistically significant.

## Results

### Macrophage PKCδ is associated with the development and progression of human atherosclerotic plaques

**Fig. 1:**
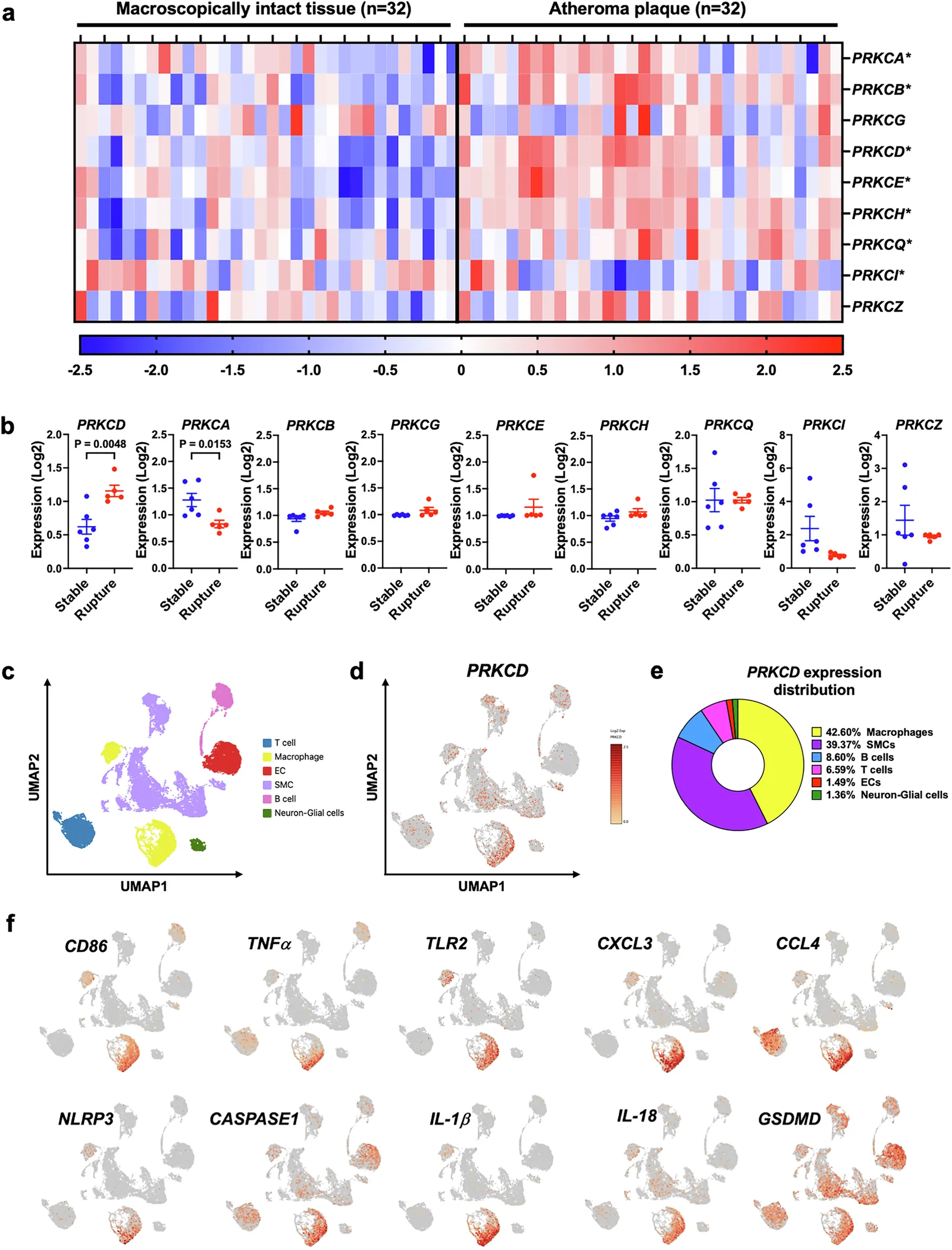
The critical role of macrophage PKCδ in human atherosclerotic plaques. **a** Gene expression analysis of PKC family genes in human atheromas compared with macroscopically intact tissue from the GSE43292 dataset. **b** Gene expression analysis of PKC family genes in macrophage-rich regions of ruptured versus stable atherosclerotic plaques from the GSE41571 dataset. **c** Uniform manifold approximation and projection (UMAP) of 43,620 atheroma cells from the GSE131778 dataset, with major cell types identified on the basis of marker gene expression. **d** Biaxial scatter plots showing the expression patterns of *PRKCD* across different subgroups within the total atheroma cell population. **e** Pie charts depicting the percentage of *PRKCD* expression in various subgroups. **f** Biaxial scatter plots showing the expression patterns of *CD68, TNFα, TLR2, CXCL3, CCL4, NLRP3, CASPASE1, IL1β, IL18, and GSDMD* across different subgroups within the total atheroma cell population. Data are expressed as mean ± s.e.m., and ^*^P < 0.05; *P* values were determined by unpaired two-tailed Student’s tests.

To investigate the role of PKCδ in atherosclerosis, we reanalyzed RNA-seq data from human carotid artery plaques and compared them with data from distant, macroscopically intact tissue (GSE43292)^16^. Our analysis revealed that the gene expression of several PKC family members, including *PRKCA*, *PRKCB*, *PRKCD*, *PRKCE*, *PRKCH*, and *PRKCQ*, was significantly upregulated in atheroma plaques, whereas *PRKCI* expression was significantly downregulated relative to the adjacent carotid tissue (Fig. 1a). To further elucidate the specific involvement of *PRKCD* in plaque vulnerability, we examined transcriptional profiles from another dataset of human carotid endarterectomy samples (GSE41571)^17^. Notably, *PRKCD* expression was significantly higher in macrophage-rich regions of ruptured atherosclerotic plaques compared with those of stable plaques (Fig. 1b). To confirm the cellular source of PKCδ within human atherosclerotic plaques, we analyzed scRNA-seq data from the GSE131778 dataset^18^ (Fig. 1c). Within atherosclerotic plaques, *PRKCD* was expressed in smooth muscle cells, B cells, and T cells, but predominantly in macrophages (Fig. 1d, e). Building on this observation, we examined the expression patterns of key inflammation-related genes within the same scRNA-seq dataset. We found that several pro-inflammatory genes, including *CD68*, *TNFα*, *TLR2*, *CXCL3*, *CCL4*, *NLRP3*, *CASPASE1*, *IL1β*, *IL18*, *and *gasdermin D (*GSDMD*)*,*were highly expressed in macrophages (Fig. 1f). This macrophage-specific enrichment of inflammatory mediators highlights their pivotal role in orchestrating immune responses within atherosclerotic plaques and supports the notion that *PRKCD* may act in concert with these pathways to regulate plaque evolution.

### Deficiency of PKCδ alleviates atherosclerosis by improving lipid profiles and restraining pro-inflammatory macrophage activity

**Fig. 2:**
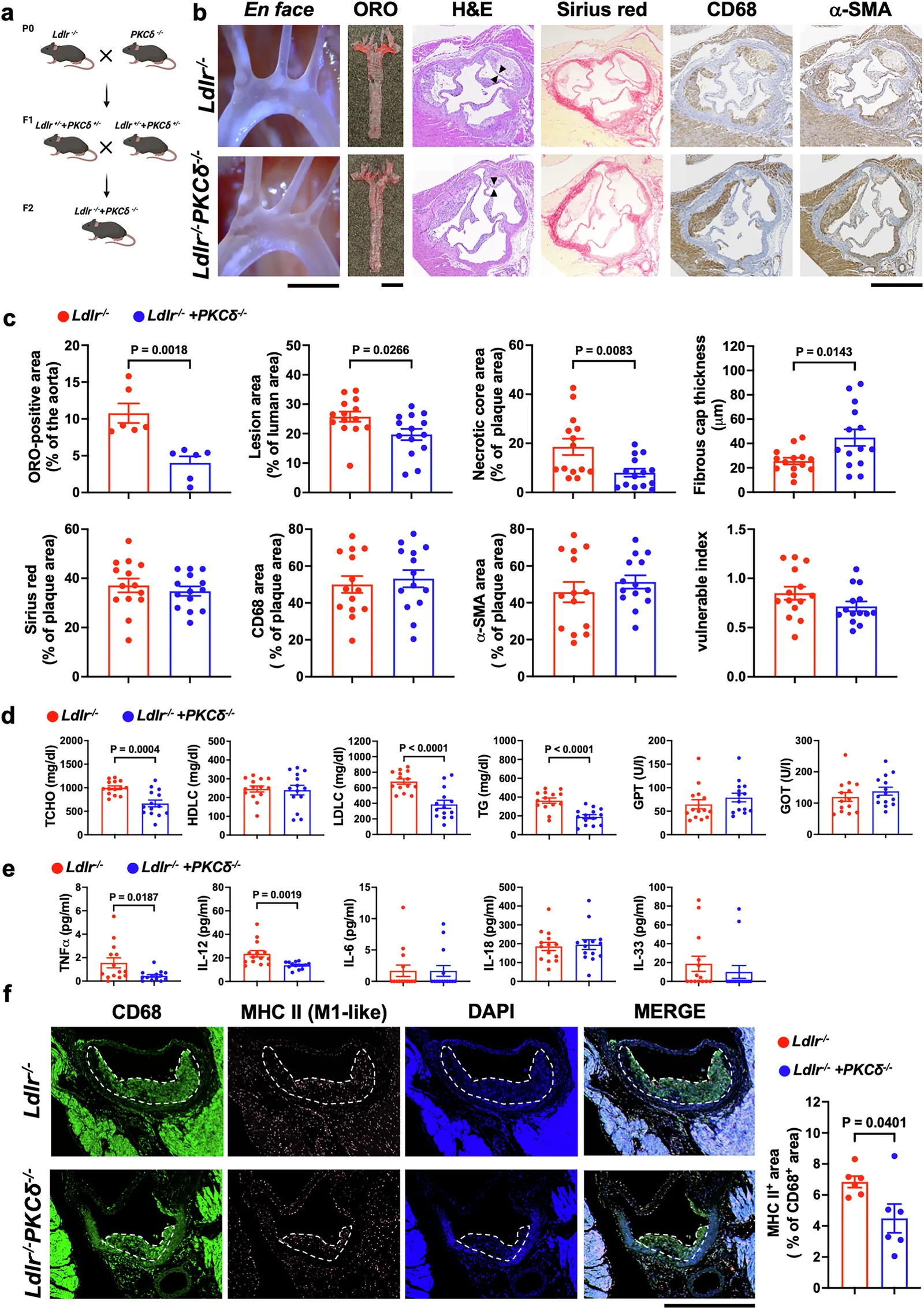
PKCδ deficiency reduces atherosclerotic plaque complexity and inflammatory responses in *Ldlr*^*-/-*^ mice. **a** Schematic diagram of the experimental strategy for *Ldlr*^*-/-*^*+PKCδ*^*-/-*^ mice breeding. **b** *Ldlr*^*-/-*^ and *Ldlr*^*-/-*^*+PKCδ*^*-/-*^ mice fed a high-cholesterol diet (HCD) for 12 weeks. Representative images of aortic arch (scale bar = 0.2 cm), oil red O (ORO; scale bar = 0.4 cm), hematoxylin and eosin (H&E), sirius red, CD68, and α-SMA staining in aortic sinus sections (scale bar = 500 μm). Black arrowheads indicate fibrous cap thickness in atherosclerotic lesions. **c** Quantification of lipid accumulation, lesion area, necrotic core, fibrous cap thickness, collagen content, macrophage content, smooth muscle cell content, and vulnerability index in the aortic sinus. (*n *= 14 in each group). **d** Plasma lipid profile of *Ldlr*^*-/-*^ and *Ldlr*^*-/-*^*+PKCδ*^*-/-*^ mice fed HCD for 12 weeks. The plasma samples were analyzed for total cholesterol (TCHO), high-density lipoprotein cholesterol (HDLC), low-density lipoprotein cholesterol (LDLC), triglycerides (TG), glutamic pyruvic transaminase (GPT), and glutamic oxaloacetic transaminase (GOT) (*n* = 14 in each group). **e** Plasma cytokine levels in *Ldlr*^*-/-*^ and *Ldlr*^*-/-*^*+PKCδ*^*-/-*^ mice fed HCD for 12 weeks. Plasma samples were analyzed using ProcartaPlex multiplex immunoassays, including TNFα, IL-12, IL-6, IL-18, and IL-33 (*n *= 14 in each group). **f** Macrophage polarization was analyzed using Opal multiplex immunofluorescence staining to identify M1-like pro-inflammatory macrophages (MHC II) among total macrophages (CD68) in aortic sinus sections from *Ldlr*^*-/-*^ and *Ldlr*^*-/-*^*+PKCδ*^*-/-*^ mice fed HCD for 12 weeks. Representative image of CD68, and MHC II staining in aortic sinus sections (scale bar = 500 μm). Quantification of the number of M1 macrophages among the total macrophages in the aortic sinus (*n* = 6 in each group). Data are expressed as mean ± s.e.m.; *P* values were determined by unpaired two-tailed Student’s *t*tests.

To elucidate the role of PKCδ in atherosclerosis, *Ldlr*^*-/-*^+*PKCδ*^*-/-*^ mice were bred (Fig. 2a) and fed HCD. En face and oil red O staining demonstrated that PKCδ deficiency significantly reduced the size of atherosclerotic plaques and lipid accumulation in the aorta (Fig. 2b, c). Hematoxylin and eosin staining revealed that PKCδ deficiency significantly decreased the lesion and necrotic core areas in the aortic sinus while increasing fibrous cap thickness on plaques (Fig. 2b, c). Further examination of plaque complexity in *Ldlr*^*-/-*^ and *Ldlr*^*-/-*^+*PKCδ*^*-/-*^ mice revealed no significant differences in macrophage infiltration, collagen content, smooth muscle cell content; however, the vulnerability index showed a trend toward reduction (Fig. 2b, c). Because hyperlipidemia is a major contributor to the initiation and progression of atherosclerosis, we analyzed plasma lipid profiles. PKCδ deficiency considerably reduced plasma total cholesterol, low-density lipoprotein cholesterol, and triglyceride levels in HCD-fed *Ldlr*^*-/-*^ mice, whereas high-density lipoprotein cholesterol and liver function indicators (GPT and GOT) remained unchanged (Fig. 2d). We then examined the impacts of PKCδ deficiency on inflammatory responses in HCD-fed *Ldlr*^*-/-*^ mice. Plasma cytokine analysis showed that PKCδ deficiency significantly reduced tumor necrosis factor alpha (TNFα) and interleukin-12 (IL-12) levels, whereas IL-18, IL-6, and IL-33 levels remained unaffected (Fig. 2e). Finally, multiplex IHC analyses revealed that PKCδ deficiency decreased the expression of MHC class II, an M1-like pro-inflammatory macrophage marker^19^, within the aortic sinus lesions of HCD-fed *Ldlr*^*-/-*^ mice (Fig. 2f).

### Hematopoietic PKCδ deficiency mitigates plaque complexity and inflammatory responses without markedly altering systemic lipid profiles

**Fig. 3:**
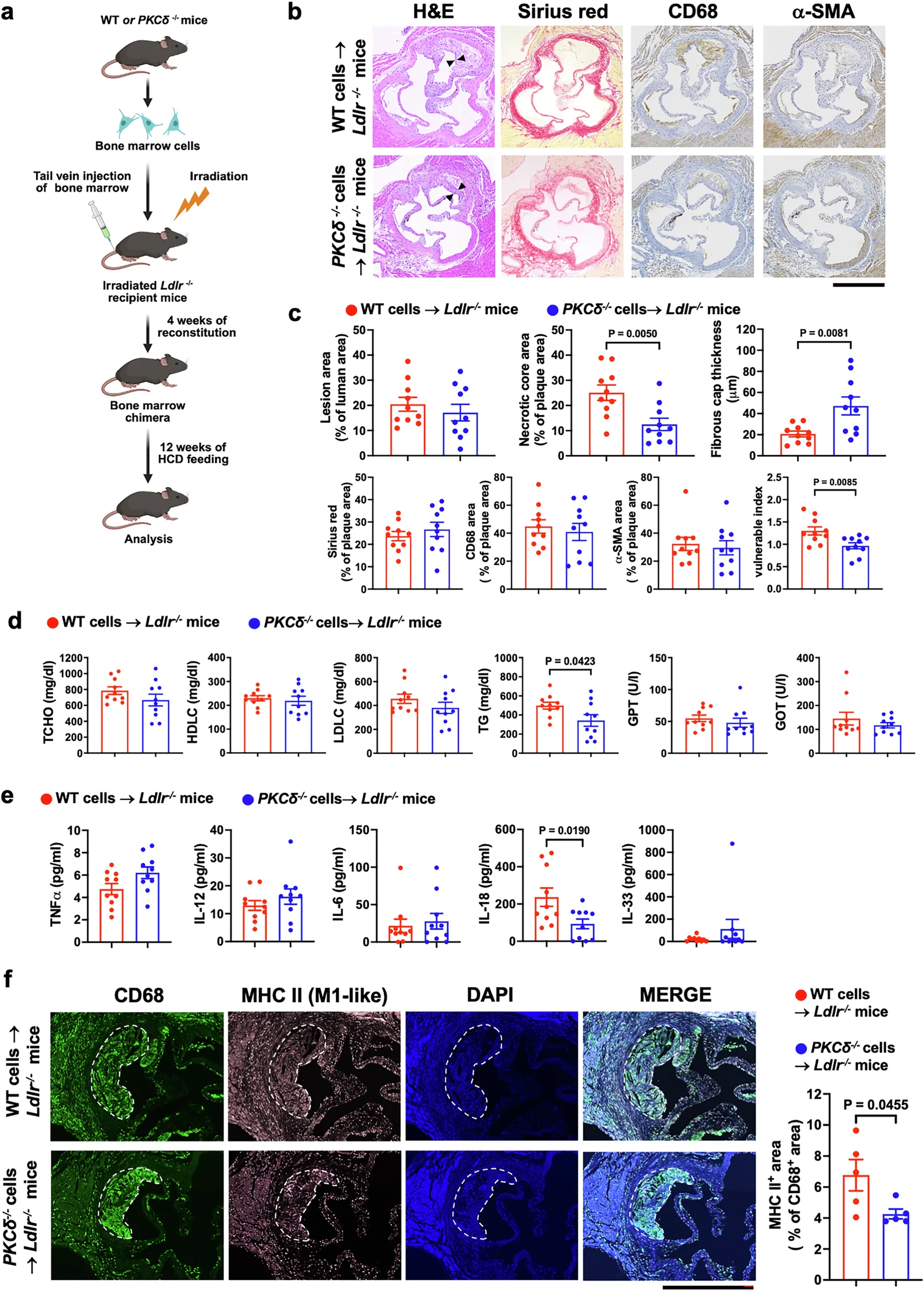
Hematopoietic PKCδ deficiency reduces atherosclerotic plaque complexity and macrophage-mediated inflammation. **a** Schematic diagram of the experimental strategy for bone marrow transplantation (BMT) and high-cholesterol diet (HCD) feeding. **b** Representative images of hematoxylin and eosin (H&E), sirius red, CD68 and α-SMA staining of aortic sinus sections from BMT mice (scale bar = 500 μm). Black arrowheads indicate fibrous cap thickness in atherosclerotic lesions. **c** Quantification of the lesion area, necrotic core, fibrous cap thickness, collagen content, macrophage content, smooth muscle cell content, and vulnerability index in the aortic sinus (*n* = 10 in each group). **d** Total cholesterol (TCHO), high-density lipoprotein cholesterol (HDLC), low-density lipoprotein cholesterol (LDLC), triglyceride (TG), glutamic pyruvic transaminase (GPT), and glutamic oxaloacetic transaminase (GOT) levels in *Ldlr*^*-/-*^ mice subjected to BMT and fed HCD for 12 weeks (*n *= 10 in each group). **e** Plasma cytokine levels in *Ldlr*^*-/-*^ mice subjected to BMT and fed HCD for 12 weeks. Plasma samples were analyzed using ProcartaPlex multiplex immunoassays, including TNFα, IL-12, IL-6, IL-18, and IL-33 (*n* = 10 in each group). **f** Representative image of CD68 and MHC II staining in aortic sinus sections from *Ldlr*^*-/-*^ mice subjected to BMT and fed HCD for 12 weeks (scale bar = 500 μm). Quantification of the number of M1 macrophages among the total macrophages in the aortic sinus (*n* = 5 in each group). Data are expressed as mean ± s.e.m.; *P* values were determined by unpaired two-tailed Student’s *t* tests.

The above data proposed that systemic PKCδ knockout affects lipid metabolism and reduces plaque burden. To specifically assess the role of hematopoietic PKCδ in atherosclerosis, *Ldlr*^*-/-*^ recipient mice underwent BMT from either WT or *PKCδ*^*-/-*^ donor mice (Fig. 3a). Hematopoietic PKCδ deficiency significantly reduced the necrotic core area and vulnerability index while increasing the thickness of the fibrous cap on plaques. There were no changes in the lesion area, macrophage infiltration, collagen content, or smooth muscle cell content (Fig. 3b, c). These findings suggest that the deficiency of hematopoietic PKCδ halts plaque evolution and reduces the complexity of atherosclerotic lesions. Furthermore, BMT from either WT or *PKCδ*^*-/-*^ mice into *Ldlr*^*-/-*^ mice for 4 weeks, followed by HCD feeding for 12 weeks, did not alter the plasma lipid profile or liver function, except for triglyceride levels (Fig. 3d). Interestingly, hematopoietic PKCδ deficiency markedly reduced the levels of the inflammatory cytokine IL-18, whereas TNFα, IL-12, IL-6, and IL-33 levels remained unchanged (Fig. 3e). Multiplexed IHC analyses further confirmed that hematopoietic PKCδ deficiency decreased the expression of MHC class II within the aortic sinus lesions (Fig. 3f). These findings suggest that PKCδ deficiency, whether systemic or restricted to hematopoietic cells, reduces plaque complexity and inflammatory responses.

### PKCδ deficiency modulates macrophage paracrine signaling to enhance fibrous cap thickness

Emerging evidence indicates that macrophage-derived paracrine factors, including transforming growth factor-β (TGFβ), platelet-derived growth factor (PDGF) and matrix metalloproteinases (MMPs), critically regulate fibrous cap formation and plaque stability by modulating extracellular matrix synthesis and degradation^20^. As shown in the Supplementary Fig. 1, under basal conditions, *PKCδ*^*-/-*^ BMDMs exhibited significantly increased TGFβ secretion compared with WT controls, whereas PDGF-B and MMP9 secretion were not affected. Upon lipopolysaccharide (LPS)/interferon-γ (IFNγ) (co-treatment with LPS and IFNγ) stimulation, TGFβ secretion was markedly suppressed, whereas PDGF-B and MMP9 secretion were increased in both WT and *PKCδ*^*-/-*^ BMDMs. Importantly, the induction of MMP9 was significantly attenuated in *PKCδ*^*-/-*^ BMDMs compared with WT controls under inflammatory conditions. Overall, these findings support a model in which PKCδ deficiency reprograms the macrophage secretory profile toward a plaque-stabilizing phenotype.

### RNA-seq analysis of the pathway by which PKCδ attenuates M1 macrophage polarization in macrophages

**Fig. 4:**
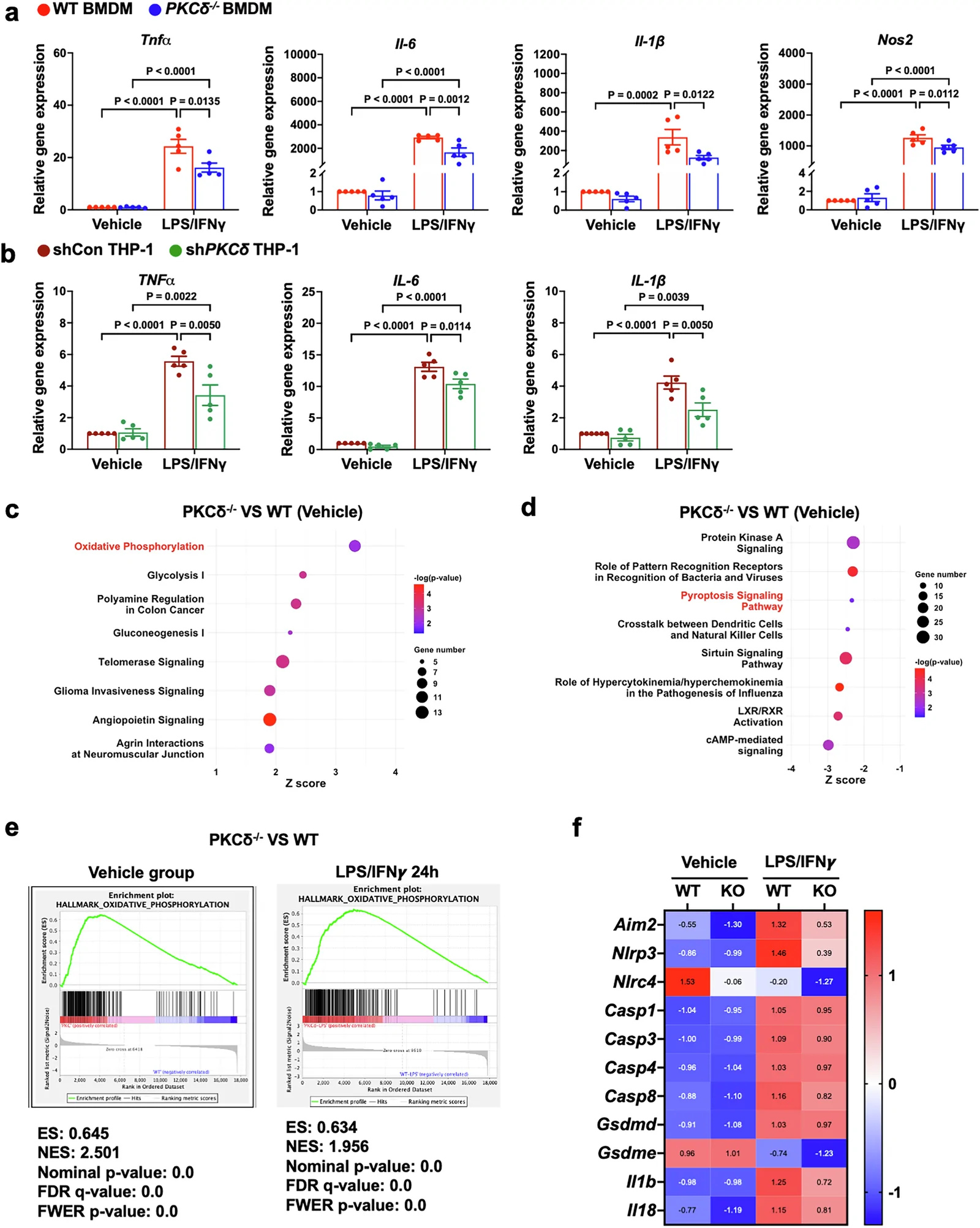
PKCδ deficiency attenuates the inflammatory pathway in vitro. **a** The gene expression levels of *Tnfα*, *Il6*, *Il1β*, and *Nos2* were measured in murine bone marrow-derived macrophages (BMDMs) isolated from wild-type (WT) or *PKCδ*^*-/-*^ mice after stimulation with lipopolysaccharide (LPS, 20 ng/ml) and interferon-γ (IFNγ, 20 ng/ml) to induce M1 macrophage-mediated inflammatory responses (*n *= 5 in each group). **b** The gene expression levels of *TNFα*, *IL6*, and *IL1β* were measured in human THP-1 cells subjected to shRNA-mediated knockdown of PKCδ, followed by stimulation with LPS (10 ng/ml) and IFNγ (10 ng/ml) to induce M1 macrophage-mediated inflammatory responses (*n* = 5 in each group). Data are expressed as mean ± s.e.m.; the *P* value was determined by two-way analysis of variance with Tukey’s test. **c** and **d** Ingenuity pathway analysis of *PKCδ*^*-/-*^ BMDMs compared with WT BMDMs. **c** A Z score > 0 indicated a positive activity pattern. **d** A Z score < 0 indicated a negative activity pattern. The red text indicates that the possible mechanism links the inflammatory responses of macrophages and necrotic core formation (*n* = 3 in each group). **e** Gene set enrichment analysis of *PKCδ*^*-/-*^ BMDMs compared with WT BMDMs under vehicle or LPS/INFγ stimulation (*n* = 3 in each group). **f** Heatmap of RNA-seq expression Z scores computed for pyroptotic-related genes between *PKCδ*^*-/-*^ and WT BMDMs under vehicle and LPS/INFγ stimulation (*n* = 3 in each group). L/I, LPS/INFγ.

To assess the effects of PKCδ on the pro-inflammatory responses of macrophages, primary bone marrow-derived macrophages (BMDMs) isolated from mice were stimulated with LPS and IFNγ to induce pro-inflammatory responses. We found that PKCδ deficiency attenuated the expression of M1 pro-inflammatory mRNAs, including *Tnfα*, *Il6*, *Il1β*, and *Nos2* (Fig. 4a), in BMDMs. In addition, the knockdown of PKCδ reduced the expression of *TNFα*, *IL6*, and *IL1β* in human THP-1 cells (Fig. 4b). To investigate the mechanism by which PKCδ attenuates pro-inflammatory responses, RNA-seq was performed to identify potential regulatory pathways. Ingenuity pathway analysis revealed that the OXPHOS pathway was the most significantly upregulated pathway (Fig. 4c). Furthermore, gene set enrichment analysis revealed that the expression of genes related to OXPHOS was significantly greater in *PKCδ*^*-/-*^ BMDMs than in WT BMDMs in both vehicle and LPS/IFNγ groups (Fig. 4e). Moreover, ingenuity pathway analysis revealed that PKCδ deficiency significantly downregulated several pathways, including protein kinase A signaling, cAMP-mediated signaling, sirtuin signaling, pyroptosis signaling pathway, the role of hypercytokinemia/hyperchemokinemia in the pathogenesis of influenza, and the role of pattern recognition receptors in the recognition of bacteria and viruses (Fig. 4d). Notably, pyroptosis is an inflammation-induced cell death mechanism involved in necrotic core formation^21^. A heatmap analysis visualizing the differential expression of pyroptosis-related genes between WT and *PKCδ*^*-/-*^ BMDMs in both vehicle and LPS/IFNγ groups (Fig. 4f) further suggested that PKCδ deficiency suppresses multiple inflammatory pathways, including pyroptosis, while enhancing OXPHOS, highlighting its key role in coordinating macrophage inflammation and metabolic regulation.

### Deficiency of PKCδ enhances immunometabolism to prevent macrophage pro-inflammatory responses

**Fig. 5:**
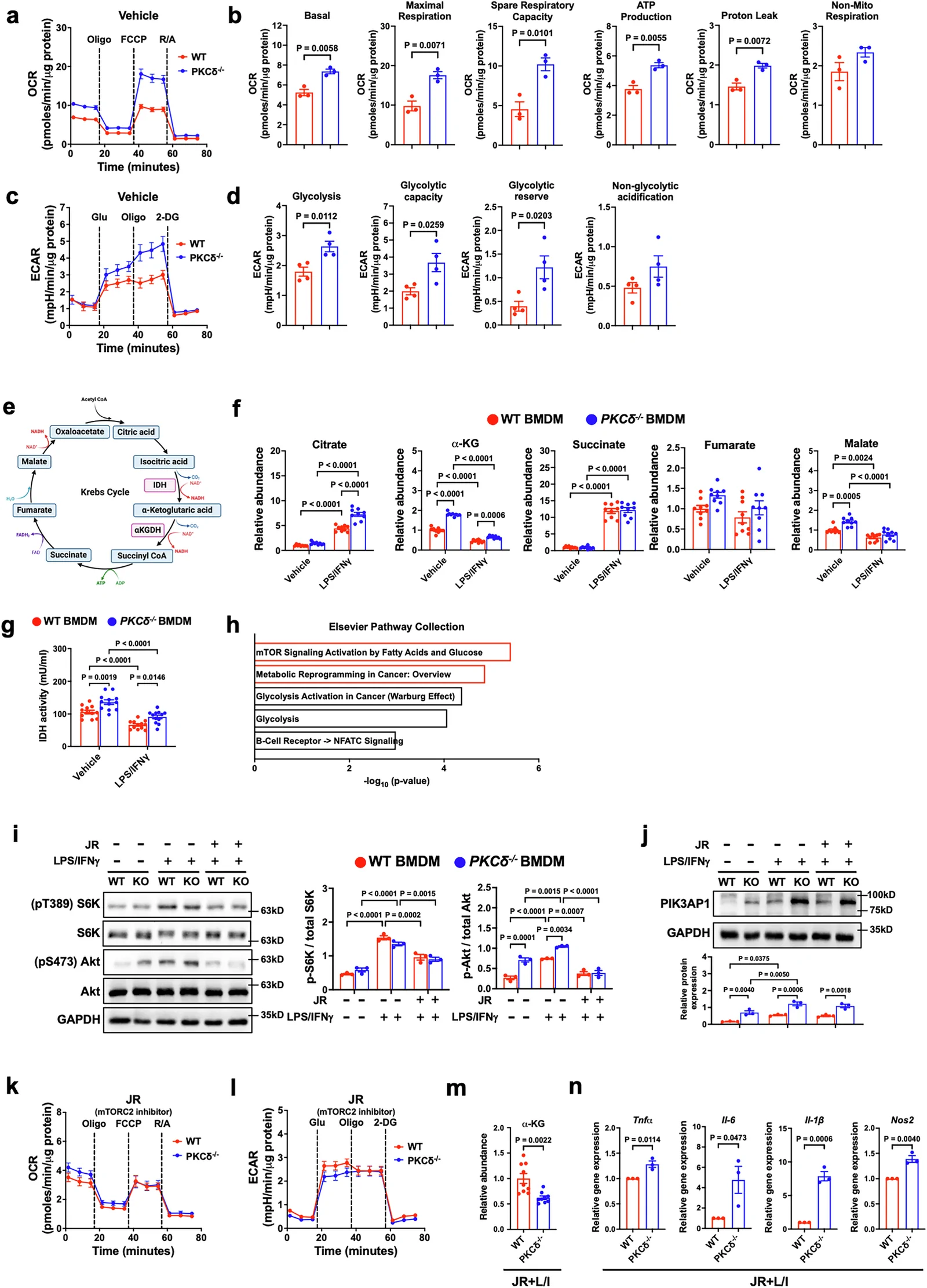
PKCδ deficiency induces changes in mitochondrial function and metabolic shifts in bone marrow-derived macrophages (BMDMs). **a** Oxygen consumption rates (OCRs) of *PKCδ*^*-/-*^ and wild-type (WT) BMDMs measured using a Seahorse analyzer. **b** Quantification of the oxidative phosphorylation (OXPHOS) parameters derived from the OCR values (*n* = 3 in each group). **c** Extracellular acidification rate (ECAR) of *PKCδ*^*-/-*^ and WT BMDMs was measured using a Seahorse analyzer. **d** Quantification of the glycolysis parameters derived from ECAR values (*n *= 4 in each group). Data are expressed as mean ± s.e.m.; *P* values were determined by unpaired two-tailed Student’s *t*tests. **e** Tricarboxylic acid (TCA) cycle schematic summarizing metabolomics. **f** The main metabolites, including citrate, α-ketoglutarate (α-KG), succinate, fumarate, and malate, were measured in *PKCδ*^*-/-*^ and WT BMDMs treated with vehicle or lipopolysaccharide (LPS)/interferon-γ (IFNγ) for 24 hours (*n *= 9 in each group). **g** Total isocitrate dehydrogenase (IDH) activity was determined in *PKCδ*^*-/-*^ and WT BMDMs treated with vehicle or LPS/IFNγ for 24 hours (*n* = 12 in each group). Data are expressed as mean ± s.e.m.; the *P* value was determined by two-way analysis of variance (ANOVA) with Tukey’s test. **h** The top five enriched pathways, ranked by *P* value, are displayed in the gene set enrichment analysis of differentially phosphorylated peptides, as shown by Enrichr bar plots (*n *= 3 per group). **i** The protein expression levels of S6K phosphorylation at T389, total S6K, Akt phosphorylation at S473, total Akt, and GAPDH were measured in BMDMs isolated from WT or *PKCδ*^*-/-*^ mice pretreated with or without JR (50 µM) for 1 hour, followed by stimulation with LPS (20 ng/ml) and IFNγ (20 ng/ml) to induce inflammatory responses (*n *= 3 in each group). **j** The protein expression levels of PIK3AP1 and GAPDH were measured in BMDMs isolated from WT or *PKCδ*^*-/-*^ mice pretreated with or without JR (50 µM) for 1 hour, followed by stimulation with LPS (20 ng/ml) and IFNγ (20 ng/ml) to induce inflammatory responses (*n* = 3 in each group). Data are expressed as mean ± s.e.m.; the *P* value was determined by two-way ANOVA with Tukey’s test. **k** OCRs of *PKCδ*^*-/-*^ and WT BMDMs treated with JR (50 µM) for 24 hours were measured using a Seahorse analyzer. **l** ECAR of *PKCδ*^*-/-*^ and WT BMDMs treated with JR (50 µM) for 24 hours, as measured using a Seahorse analyzer. **m** α-KG levels were measured in *PKCδ*^*-/-*^ and WT BMDMs pretreated with JR (50 µM) for 1 hour, followed by stimulation with LPS/IFNγ for 24 hours (*n *= 9 in each group). **n** The gene expression levels of *Tnfα*, *Il6*, *Il1β*, and *Nos2* were measured in BMDMs isolated from WT or *PKCδ*^*-/-*^ mice pretreated with JR (50 µM) for 1 hour, followed by stimulation with LPS (20 ng/ml) and IFNγ (20 ng/ml) to induce M1 macrophage-mediated inflammatory responses (*n* = 3 in each group). Data are expressed as mean ± s.e.m.; *P* values were determined by unpaired two-tailed Student’s *t*tests. L/I, LPS/IFNγ. Oligo, oligomycin; FCCP, carbonyl cyanide-p-trifluoromethoxyphenylhydrazone; R/A, rotenone/antimycin; Glu, glucose; 2-DG, 2-deoxy-D-glucose; JR, JR-AB2-011.

During the pro-inflammatory responses of macrophages, mitochondrial fitness was decreased, which involves the suppression of OXPHOS and a shift toward glycolysis for ATP production^8^. Our RNA-seq analysis indicated that the OXPHOS pathway is enriched in *PKCδ*^*-/-*^ BMDMs. These findings prompted us to explore the role of PKCδ in macrophage metabolism. We found that PKCδ deficiency enhanced OXPHOS (Fig. 5a, b) and glycolytic activity (Fig. 5c, d). Under LPS/IFNγ stimulation, compared with WT BMDMs, *PKCδ*^*-/-*^ BMDMs tended toward increased OXPHOS (Supplementary Fig. 2a, b) and glycolysis (Supplementary Fig. 2c, d). An energy map revealed that LPS/IFNγ stimulation shifted the metabolic phenotype toward glycolysis, but *PKCδ*^*-/-*^ BMDMs exhibited a more energetic phenotype in both vehicle-treated and LPS/IFNγ-stimulated groups (Supplementary Fig. 2e). TCA cycle metabolites are crucial for mitochondrial fitness by regulating OXPHOS and play an important role in the pro-inflammatory responses of macrophages. We then investigated the effects of PKCδ on TCA cycle metabolism (Fig. 5e) in WT and *PKCδ*^*-/-*^ BMDMs using ultra-performance liquid chromatography triple quadrupole mass spectrometry. Moreover, PKCδ deficiency in BMDMs induced broad metabolic reprogramming, with most metabolites showing an upward trend; notably, citrate and α-KG levels remained persistently elevated compared with WT BMDMs, even under LPS/IFNγ stimulation (Fig. 5f). Furthermore, total isocitrate dehydrogenase (IDH) activity was significantly increased in *PKCδ*^*-/-*^ BMDMs (Fig. 5g). The increased abundance of TCA cycle metabolites suggests enhanced mitochondrial activity, which may reflect improved mitochondrial fitness.

Next, we conducted a phosphoproteomic analysis to evaluate the effects of PKCδ deficiency on the activity of other kinases. Kinase–substrate enrichment analysis based on phosphorylated peptide levels revealed a reduced activity of several PKC family members in *PKCδ*^*-/-*^ BMDMs. In addition, inflammation-related kinases such as IRE1, NEK2, PDK1, and RIPK2 showed decreased activity (Supplementary Fig. 3a). Consistently, upon LPS/IFNγ stimulation, the activities of inflammation-related kinases, including HRI, IRAK4, LOK, NEK2, PDK1, and RIPK2, were markedly lower in *PKCδ*^*-/-*^ BMDMs than in WT BMDMs (Supplementary Fig. 3b). Among these, IRAK4 and RIPK2 are pivotal in TLR/IL-1R and NOD-mediated NF‑κB activation, driving pro‑inflammatory cytokine production^22,23^. Phosphoproteomic analysis of BMDMs revealed significant changes in phosphorylation across ten distinct clusters (Supplementary Fig. 3c). Interestingly, clusters 1 and 2 contained 121 peptides with higher phosphorylation levels in *PKCδ*^*-/-*^ BMDMs than in WT BMDMs. Enrichment analysis of phosphoproteomic data from these clusters highlighted pathways such as “mTOR Signaling Activation by Fatty Acids and Glucose,” and “Metabolic Reprogramming in Cancer: Overview.” The enrichment of mTOR signaling and cancer-related metabolic reprogramming pathways indicates that PKCδ deficiency promotes lipid- and glucose-driven metabolic reprogramming, shifting macrophages toward a glycolytic and metabolically active state that may influence inflammatory responses. These findings suggest that mTOR signaling acts as a central regulator of metabolic remodeling in *PKCδ*^*-/-*^ BMDMs (Fig. 5h). To elucidate how mTORC controls mitochondrial function in PKCδ-deficient macrophages, we examined phosphorylation patterns and found that PKCδ deficiency increased Akt S473 phosphorylation but not p70 S6 kinase (S6K) T389 phosphorylation, indicating the selective activation of mTORC2 rather than mTORC1 (Fig. 5i). To further investigate the potential upstream regulators of mTORC2, we examined targets enriched in clusters 1 and 2 by phosphoproteomic analysis (Supplementary Fig. 3c) and demonstrated that phosphoinositide-3-kinase adaptor protein 1 (PIK3AP1), a previously reported upstream regulator of Akt Ser473 phosphorylation^24^, was significantly upregulated in the absence of PKCδ. Moreover, our data confirmed that *Pik3ap1* mRNA expression was significantly increased in *PKCδ*^*-/-*^ BMDMs (Supplementary Fig. 3d), suggesting that the upregulation of PIK3AP1 was regulated at the transcriptional level. We further showed that PIK3AP1 expression was enhanced in *PKCδ*^*-/-*^ BMDMs via the Akt-independent pathway (Fig. 5j). Treatment with the mTORC2 inhibitor JR-AB2-011 (JR) eliminated the phosphorylation of Akt S473 (Fig. 5i) and normalized both OXPHOS and glycolysis in *PKCδ*^*-/-*^ and WT BMDMs (Fig. 5k, l) but did not affect the increased protein expression of PIK3AP1 (Fig. 5j). Similarly, JR treatment reduced α-KG levels in *PKCδ*^*-/-*^ BMDMs (Fig. 5m). Furthermore, mTORC2 inhibition reversed the suppression of the pro-inflammatory responses of macrophages caused by PKCδ deficiency (Fig. 5n). These findings establish mTORC2 as the key mediator of PKCδ-dependent metabolic reprogramming during the pro-inflammatory responses of macrophages. Our findings indicate that PKCδ deficiency enhances mitochondrial fitness, promoting OXPHOS and significantly altering TCA cycle metabolism while increasing glycolytic capacity. This metabolic reprogramming occurs through PIK3AP1–mTORC2 activation, positioning PKCδ as a potential therapeutic target for modulating macrophage function under inflammatory conditions.

### PKCδ deficiency attenuates the inflammasome and pyroptosis in macrophages

**Fig. 6:**
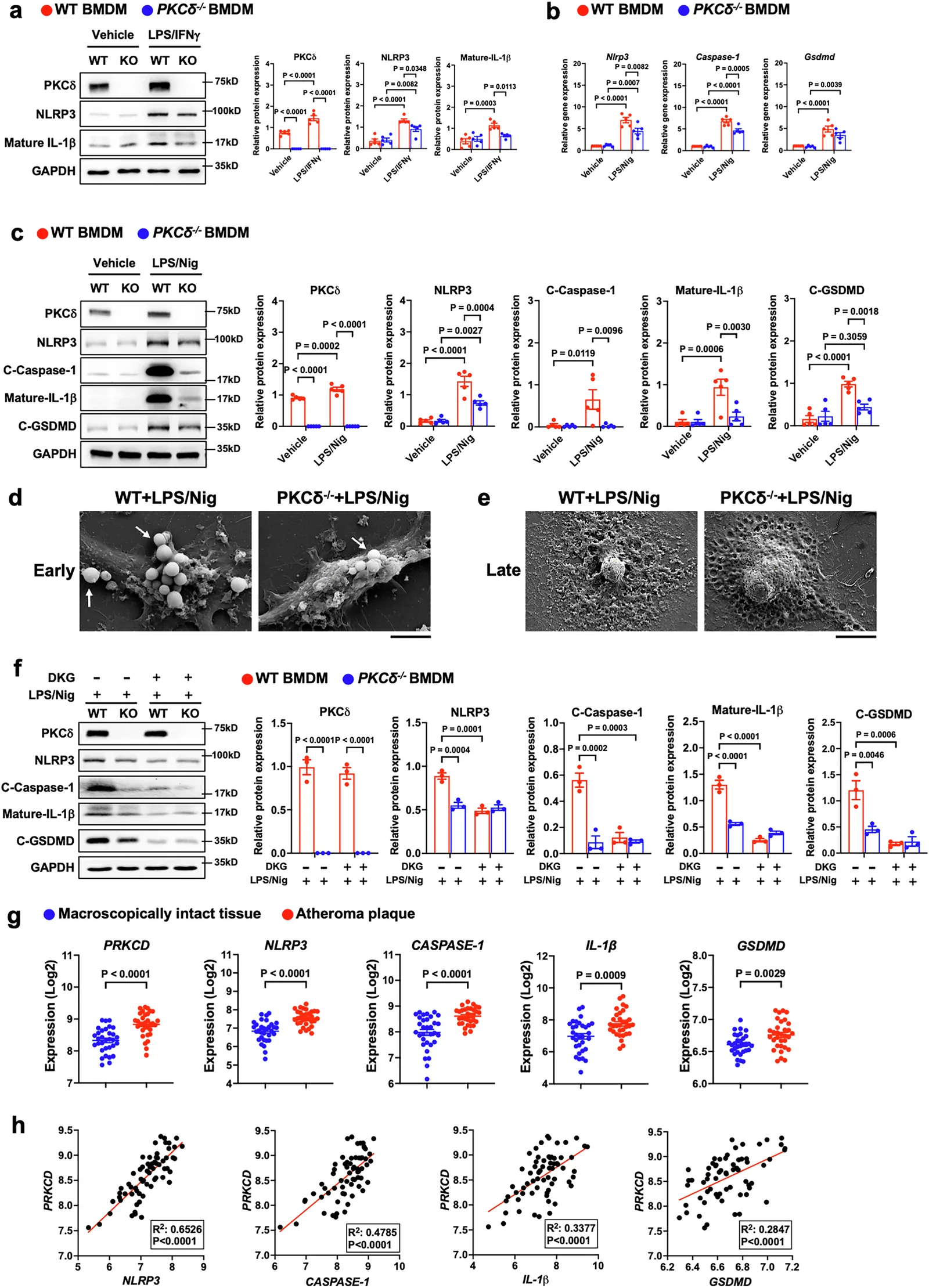
PKCδ deficiency reduces NLRP3 inflammasome-mediated pyroptosis. **a** The protein expression of PKCδ, NLRP3, and IL-1β was measured in bone marrow-derived macrophages BMDMs isolated from wild-type (WT) or *PKCδ*^*-/-*^ mice after stimulation with lipopolysaccharide (LPS, 20 ng/ml) and interferon-γ (IFNγ, 20 ng/ml) to induce the inflammasome (*n *= 5 in each group). **b** The gene expression levels of *Nlrp3*, *Casp1*, and *Gsdmd* were measured in BMDMs isolated from WT or *PKCδ*^*-/-*^ mice after stimulation with LPS (100 ng/ml) for 3 hours and Nig (1 μM) for 1 hour to induce pyroptosis (n = 5 in each group). **c** The protein expression levels of PKCδ, NLRP3, cleaved caspase 1, mature IL-1β, and cleaved GSDMD were measured in BMDMs isolated from WT or *PKCδ*^*-/-*^ mice following stimulation with LPS (100 ng/ml) for 3 hours and nigericin (Nig, 1 μM) for 1 hour to induce pyroptosis (*n *= 5 in each group). Data are expressed as mean ± s.e.m.; the *P* value was determined by two-way analysis of variance (ANOVA) with Tukey’s test. **d** Representative scanning electron microscopy (SEM) images of early pyroptosis in WT and *PKCδ*^*-/-*^ BMDMs. The arrows indicate pyroptotic bodies (*n *= 3 in each group). Scale bar = 10 μm. **e** Representative SEM images of late pyroptosis in WT and *PKCδ*^*-/-*^ BMDMs (*n *= 3 in each group). Scale bar = 10 μm. **f** The protein expression levels of PKCδ, NLRP3, cleaved caspase 1, mature IL-1β, and cleaved GSDMD were measured in BMDMs isolated from WT or *PKCδ*^*-/-*^ mice pretreated with dimethyl α-ketoglutarate (DKG, 5 mM) for 1 hour, followed by stimulation with LPS (100 ng/ml) for 3 hours and Nig (1 μM) for 1 hour to induce pyroptosis (*n* = 3 in each group). Data are expressed as mean ± s.e.m.; the *P* value was determined by two-way ANOVA with Tukey’s test. **g** Gene expression analysis of *PRKCD*, *NLRP3*, *CASP1*, *IL1β*, and *GSDMD* in human atheromas compared with macroscopically intact tissue from the GSE43292 dataset (*n* = 32 in each group). Data are expressed as mean ± s.e.m.; *P* values were determined by unpaired two-tailed Student’s *t*tests. **h** Simple linear regression analysis between *PRKCD* expression and *NLRP3*, *CASP1*, *IL1β*, or *GSDMD* expression in human carotid endarterectomy samples from the GSE43292 dataset (*n *= 64). KO, knockout; KD, knockdown; C-Caspase 1, cleaved caspase 1; C-GSDMD, cleaved GSDMD.

Mitochondrial dysfunction and metabolites activate the NLRP3 inflammasome, triggering pyroptotic cell death that contributes to necrotic core formation in atherosclerotic plaques^25^. Our RNA-seq data revealed that PKCδ deficiency suppresses pyroptosis pathway activation, prompting us to investigate the role of PKCδ in regulating inflammasome-mediated pyroptosis. Under LPS/IFNγ stimulation, the expression of PKCδ significantly increased in both BMDMs and THP-1 cells (Fig. 6a and Supplementary Fig. 4a). PKCδ deficiency decreased the protein expression of NLRP3 and mature IL-1β in BMDMs and THP-1 cells treated with LPS/IFNγ (Fig. 6a and Supplementary Fig. 4a). LPS combined with nigericin (Nig) is commonly used to trigger pyroptosis via canonical inflammasome activation. Compared with WT BMDMs, *PKCδ*^*-/-*^ BMDMs exhibited reduced mRNA expression of *Nlrp3* and *Caspase 1* (*Casp1*) upon LPS/Nig stimulation (Fig. 6b), whereas the mRNA levels of *NLRP3*, *CASP1*, and *GSDMD* remained unchanged in PKCδ-knockdown THP-1 human macrophages compared with controls (Supplementary Fig. 4b). Our data demonstrated that PKCδ deficiency reduced protein expression of NLRP3, mature IL-1β, cleaved GSDMD, and active caspase 1 in both BMDMs and THP-1 cells treated with LPS/Nig (Fig. 6c and Supplementary Fig. 4c). Morphological analysis by scanning electron microscopy revealed that pyroptosis features, including pyroptotic bodies and pores, were substantially lower in *PKCδ*^*-/-*^ BMDMs than in WT BMDMs (Fig. 6d, e).

Using both computational prediction tools and biochemical analysis, we examined the potential interaction between PKCδ and NLRP3 (Supplementary Fig. 5). AlphaFold Multimer analysis yielded low interface confidence scores (ipTM = 0.17, pTM = 0.33) (Supplementary Fig. 5a), and PEPPI analysis predicted a low likelihood of interaction (log(LR) = −1.328) (Supplementary Fig. 5b). Consistently, co-immunoprecipitation assays failed to detect an association between PKCδ and NLRP3 under either basal or LPS/Nig-stimulated conditions in BMDMs (Supplementary Fig. 5c). Notably, PKCδ deficiency significantly reduced *Nlrp3* mRNA expression after LPS/Nig stimulation (Fig. 6c). Collectively, these data indicate that the reduction of NLRP3 and cleaved caspase 1 in *PKCδ*^*-/-*^ BMDMs is more likely mediated by indirect regulatory mechanisms rather than direct interaction or phosphorylation of NLRP3 by PKCδ.

As mentioned above, PKCδ deficiency increases α-KG production, which may enhance mitochondrial fitness by sustaining TCA cycle flux, supporting OXPHOS, maintaining redox balance, and promoting metabolic flexibility^26^. To assess the role of α-KG-supported mitochondrial fitness in pyroptosis, BMDMs were treated with the cell-permeable α-KG analog DKG. We found that DKG supplementation reduced the expression of pyroptosis-related protein, including NLRP3, active caspase 1, mature IL-1β, and cleaved GSDMD in both WT and *PKCδ*^*-/-*^ BMDMs (Fig. 6f). Our data suggest that α-KG functions as a downstream effector of PKCδ, suppressing pyroptosis and linking PKCδ deficiency to reduced inflammasome activation through enhanced mitochondrial function.

**Fig. 7:**
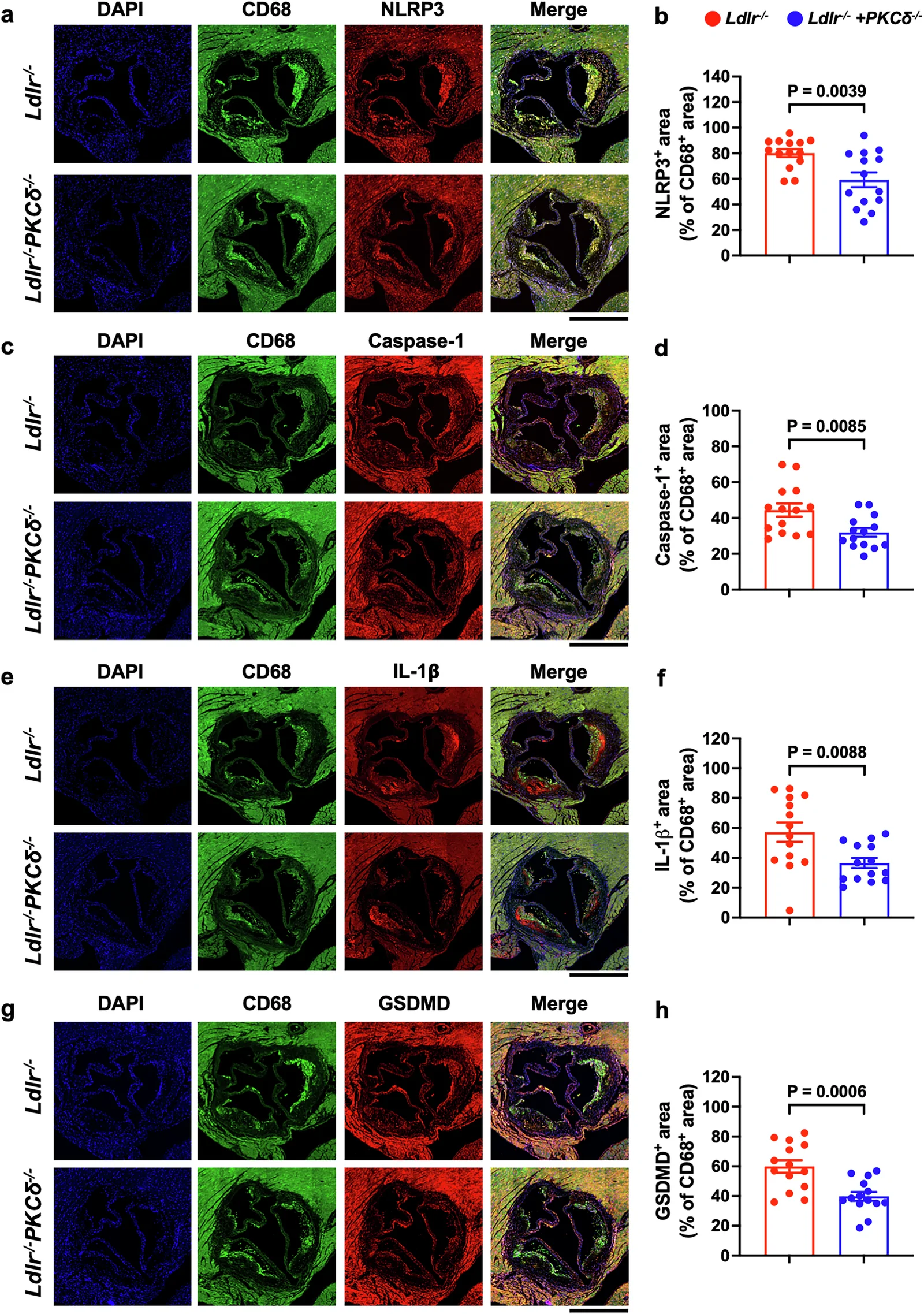
PKCδ deficiency reduces NLRP3 inflammasome-mediated pyroptosis in atherosclerotic plaques in the aortas of *Ldlr*^*-/-*^ mice fed high-cholesterol diet (HCD). **a** Representative immunofluorescence image of NLRP3 and CD68 co-staining in the aortic sinuses of HCD-fed *Ldlr*^*-/-*^ and *Ldlr*^*-/-*^*+PKCδ*^*-/-*^ mice. **b** Quantification of the area in which NLRP3 and CD68 were co-localized in the aortic sinus of HCD-fed *Ldlr*^*-/-*^ mice (*n *= 14 in each group). **c** Representative immunofluorescence image of caspase 1 and CD68 co-staining in the aortic sinuses of HCD-fed *Ldlr*^*-/-*^ and *Ldlr*^*-/-*^*+PKCδ*^*-/-*^ mice. **d** Quantification of the area in which caspase 1 and CD68 were co-localized in the aortic sinuses of HCD-fed *Ldlr*^*-/-*^ and *Ldlr*^*-/-*^*+PKCδ*^*-/-*^mice (*n *= 14 in each group). **e** Representative immunofluorescence image of IL-1β and CD68 co-staining in the aortic sinuses of HCD-fed *Ldlr*^*-/-*^ and *Ldlr*^*-/-*^*+PKCδ*^*-/-*^ mice. **f** Quantification of IL-1β and CD68 co-localization in the aortic sinus of HCD-fed *Ldlr*^*-/-*^ mice (*n *= 14 in each group). **g** Representative immunofluorescence image of GSDMD and CD68 co-staining in the aortic sinuses of HCD-fed *Ldlr*^*-/-*^ and *Ldlr*^*-/-*^*+PKCδ*^*-/-*^ mice. **h** Quantification of GSDMD and CD68 co-localization in the aortic sinuses of HCD-fed *Ldlr*^*-/-*^ and *Ldlr*^*-/-*^*+PKCδ*^*-/-*^ mice (*n *= 14 in each group). Data are expressed as mean ± s.e.m.; *P* values were determined by unpaired two-tailed Student’s *t*tests. Scale bar = 500 μm.

**Fig. 8:**
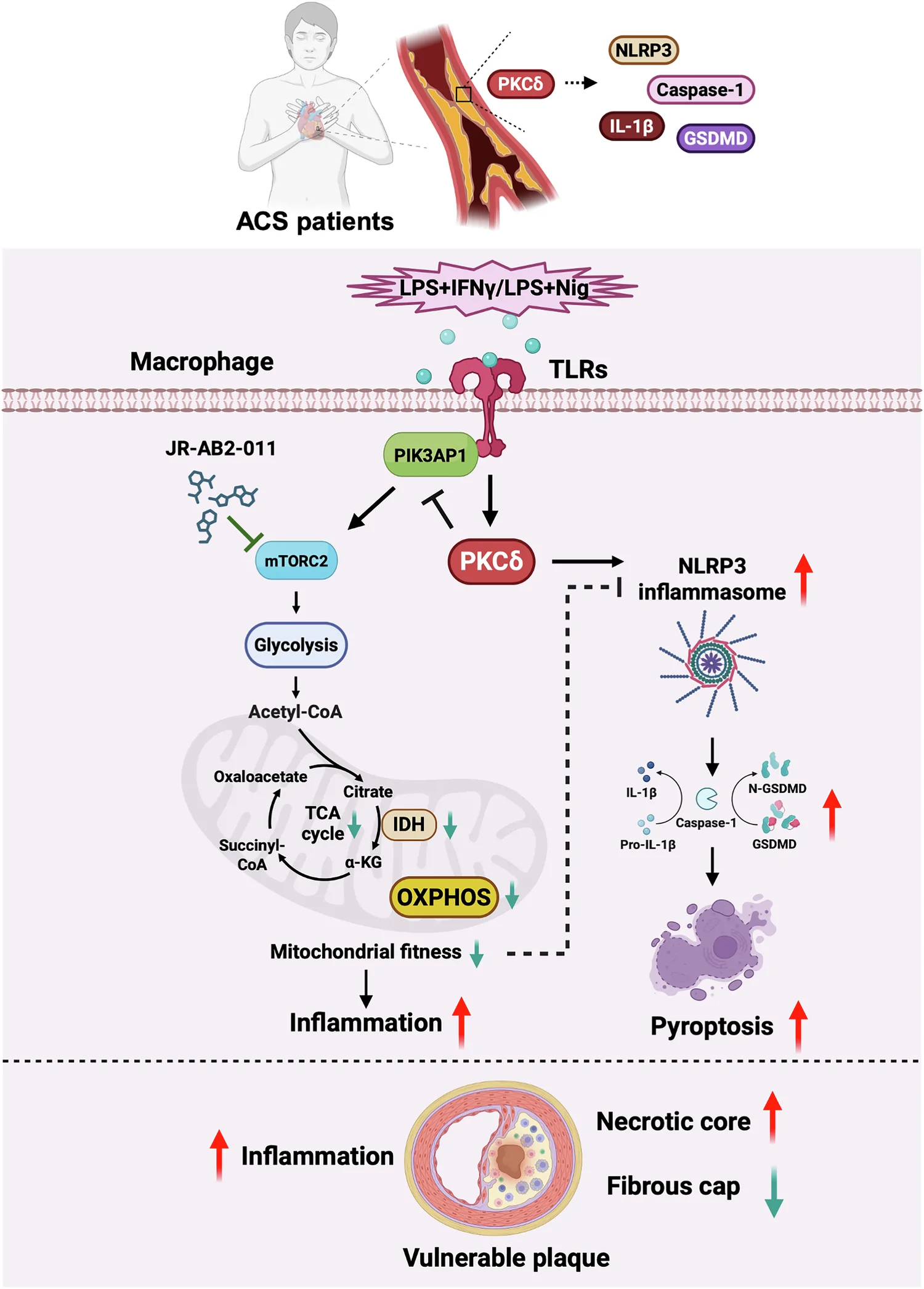
PKCδ-mediated regulation of macrophage metabolism and NLRP3 inflammasome determines atherosclerotic plaque stability. The schematic shows that PKCδ deficiency improves atherosclerotic plaque stability. PKCδ likely plays a key role in regulating the NLRP3 inflammasome in the atheromas of patients with acute coronary syndrome (ACS). PKCδ is essential for regulating M1 macrophage-mediated inflammatory responses and acts by modulation of glycolysis and the tricarboxylic acid (TCA) cycle. PKCδ influences the mitochondrial metabolite α-ketoglutarate (α-KG), and is involved in NLRP3 inflammasome-mediated pyroptosis, which contributes to necrotic core formation. Mechanistically, PKCδ promotes inflammasome activation and pyroptosis by inhibiting the PIK3AP1–mTORC2–α-KG pathway. These findings highlight the central role of PKCδ in plaque evolution and acute cardiovascular events. LPS, lipopolysaccharide; IFNγ, interferon-γ; Nig, nigericin; OXPHOS, oxidative phosphorylation; IDH, isocitrate dehydrogenase.

Mitochondrial reactive oxygen species (mtROS) is a well-established upstream trigger for NLRP3 inflammasome activation, facilitating ASC oligomerization and subsequent caspase 1 activation^25^. To determine whether PKCδ deficiency regulates inflammasome activation through modulation of redox homeostasis, we assessed mtROS production in BMDMs. After LPS/IFNγ stimulation, *PKCδ*^*-/-*^ BMDMs exhibited significantly reduced mtROS production compared with WT BMDMs. Importantly, exogenous DKG treatment further suppressed mtROS levels under the same conditions (Supplementary Fig. 6). Our findings provide mechanistic support that α-KG suppresses inflammasome activation, in part, by attenuating mtROS-dependent signaling. Reanalysis of RNA-seq data from human carotid artery plaques compared with those from distant, macroscopically intact tissue (GSE43292)^16^ revealed significantly increased expression of *PRKCD*, *NLRP3*, *NLRC4, AIM2*, *IL1β*, *CASP1*, and *GSDMD* in human atheromas (Fig. 6g and Supplementary Fig. 7a). Correlation analysis revealed that *PRKCD* expression was positively correlated with the expression of *NLRP3*, *NLRC4, AIM2*, *IL1β*, *CASP1*, and *GSDMD* (Fig. 6h and Supplementary Fig. 7b). Notably, Pearson’s correlation analysis indicated that *NLRP3* expression showed the strongest correlation with *PRKCD* expression in human atheromas (Supplementary Fig. 7c). Interestingly, plasma PKCδ levels were comparable between patients with ACS and those with stable angina (Supplementary Fig. 8a). To further assess the impacts of PKCδ on pyroptosis in plaque macrophages from HCD-fed *Ldlr*^*-/-*^ mice, we conducted immunofluorescence staining. PKCδ deficiency reduced the co-localization of CD68 with canonical pyroptosis markers (NLRP3, caspase 1, IL-1β, and GSDMD) in plaques of HCD-fed *Ldlr*^*-/-*^ mice (Fig. 7a–h). These data suggest that PKCδ regulates the NLRP3 inflammasome and pyroptosis in macrophages, contributing to necrotic core formation in atherosclerosis. The proposed working models of PKCδ in atherosclerosis are summarized in Fig. 8.

## Discussion

In this study, we examined the role of PKCδ in atherosclerosis, focusing on its impact on macrophage polarization, pyroptosis, and plaque evolution. Elevated PKCδ expression in macrophage-rich areas of ruptured human plaques, together with findings that PKCδ deficiency reduced plaque size, lipid accumulation, and necrotic core areas in *Ldlr*^*-/-*^ mice, indicates that PKCδ is critical for plaque evolution. Mechanistically, PKCδ deficiency enhanced OXPHOS and glycolysis in macrophages, modulating metabolic and energy pathways via PIK3AP1–mTORC2 activation to suppress pro-inflammatory responses of macrophages. Moreover, PKCδ deficiency suppressed pyroptosis and attenuated necrotic core formation, potentially through the upregulation of α-KG. These findings establish PKCδ inhibition as a promising therapeutic strategy for stabilizing vulnerable plaques.

Omics data, including scRNA-seq and bulk RNA-seq, revealed that PKCδ is highly expressed in macrophages within human atherosclerotic plaques, particularly ruptured plaques. Importantly, we detected a positive correlation between PKCδ and its key associated molecules, such as NLRP3, caspase 1, GSDMD, and IL-1β, which contribute to necrotic core formation. The CANTOS study, which demonstrated reduced major adverse cardiovascular events through IL-1β blockade^2^, has heightened interest in targeting inflammation in cardiovascular disease. Considering molecules currently under investigation in clinical trials^27^, PKCδ emerges as a promising therapeutic target for future cardiovascular interventions.

Interestingly, PRKCI expression was significantly downregulated in atheroma plaques compared with adjacent carotid tissues in bulk RNA-seq data. The current evidence regarding the role of PKCι (PRKCI) in atherosclerosis remains limited and somewhat inconsistent. One study reported that saturated fatty acids can activate PKCι and mediate lipid-induced apoptosis in endothelial cells, suggesting a potential role in endothelial injury during atherosclerosis development^28^. Conversely, another study demonstrated that inflammatory stimuli such as C-reactive protein and endothelin 1 inhibit PKCλ/ι translocation and activity, leading to impaired nitric oxide production and endothelial dysfunction^29^. Taken together, these findings suggest that the role of PKCι in atherosclerosis may be context-dependent and remains incompletely understood. Additional studies are therefore needed to clarify its precise contribution to vascular inflammation and plaque development.

Macrophages exhibit time-dependent behavior along the inflammatory-reparative spectrum during immune responses. After LPS/IFNγ stimulation, the pro-inflammatory phase predominates for 16–24 hours before transitioning to a reparative phase^30^. This two-stage metabolic remodeling prevents sustained inflammation by shifting macrophages from pro-inflammatory to M2-like reparative phenotypes that rely on the TCA cycle, fatty acid oxidation, and OXPHOS for energy production^31,32^. Previous studies have shown that mTORC2 mediates glutaminolysis, which converts glutamine to α-KG to replenish TCA cycle intermediates and maintain mitochondrial function^33,34^. Our data indicate that PKCδ deficiency maintains mitochondrial fitness by increasing TCA cycle metabolites and OXPHOS activity, resulting in reduced inflammation. These findings suggest that PKCδ deficiency enhances the reparative phase by promoting anti-inflammatory metabolic reprogramming, thereby facilitating the resolution of inflammation.

Accumulating evidence indicates that metabolic reprogramming is a key determinant of macrophage inflammatory phenotypes. In classically activated M1 macrophages, the TCA cycle is functionally disrupted at critical nodes, particularly IDH and succinate dehydrogenase, leading to the accumulation of pro-inflammatory metabolites. IDH inhibition results in citrate accumulation, which fuels TNFα and prostaglandin production^35^, whereas succinate dehydrogenase inhibition leads to succinate accumulation and ROS production, both of which stabilize HIF1α and enhance IL-1β production^36^. Interestingly, TCA intermediates might exert anti-inflammatory effects depending on the metabolic context. Citrate-derived itaconate suppresses IL-1β, IL-6, and HIF1α signaling^37^, whereas α-KG supports OXPHOS and promotes M2-like polarization through epigenetic regulation, alongside inhibiting LPS/IFNγ-induced NF-κB and NLRP3 expression^38,39^. Our data suggest that PKCδ deficiency preserves TCA cycle integrity by maintaining key enzyme activity (e.g., IDH), thereby limiting pro-inflammatory metabolite accumulation and sustaining mitochondrial function through mTORC2. This metabolic preservation promotes a shift away from an M1-like state toward a more restrained, metabolically balanced phenotype. Together, our findings support a model in which PKCδ deficiency attenuates macrophage inflammation by maintaining TCA cycle homeostasis and reprogramming immunometabolic signaling.

Our data revealed that PKCδ deficiency decreases necrotic core formation and strengthens the fibrous cap of atherosclerotic plaques in HCD-fed *Ldlr*^*-/-*^ mice, prompting us to investigate the underlying cellular mechanisms involved. RNA-seq analysis revealed marked suppression of the pyroptosis pathway. Canonical pyroptosis is mediated by inflammasome-dependent activation of caspase 1, which cleaves pro-IL-1β, pro-IL-18, and GSDMD, facilitating cytokine release and inducing pyroptotic cell death^21^. Our in vitro and in vivo results demonstrated that PKCδ deficiency markedly suppressed NLRP3, caspase 1, IL-1β, and GSDMD activation in LPS/Nig-treated BMDMs and reduced their expression in the aortic sinuses of HCD-fed *Ldlr*^*-/-*^ mice. The mechanism underlying PKCδ-mediated regulation of pyroptosis involves metabolic reprogramming through the mTORC2–α-KG axis. We established that PKCδ deficiency activates mTORC2, leading to increased α-KG production, an effect that is reversed by mTORC2 inhibition, confirming pathway specificity. This metabolic shift toward elevated α-KG levels provides multiple antipyroptotic benefits. Notably, α-KG has been reported to inhibit NLRP3-mediated pyroptosis in human ovarian granulosa cells^39^. Moreover, α-KG enhances mitochondrial quality control by promoting mitophagy and improving mitochondrial structure and function while simultaneously reducing ROS production^26^. Our results indicate that α-KG attenuates mitochondrial oxidative stress in BMDMs and suggest that PKCδ regulates NLRP3-driven pyroptotic signaling through α-KG-mediated metabolic reprogramming.

Our data suggest that mTORC2 signaling contributes to the antiatherosclerotic effects of PKCδ deficiency. PKCδ deficiency enhances mTORC2-dependent Akt phosphorylation, glycolysis, OXPHOS, and α-KG levels, collectively inhibiting M1 macrophage polarization. Charbonnier et al. reported that loss of mTORC2 activity impairs glycolysis and OXPHOS in regulatory T cells^40^. Moreover, mTORC2 plays a key role in atherosclerosis by regulating macrophage function and suppressing pro-inflammatory pathways, including inflammasome/IL-1β response. In macrophage-specific mTORC2-deficient mice, increased lesion complexity and cell death further suggest that mTORC2 has a protective role in controlling inflammation and promoting cell survival^41^. Our study identifies a novel link between PKCδ and PIK3AP1, demonstrating for the first time that PKCδ deficiency upregulates PIK3AP1 protein expression. PIK3AP1 reportedly regulates Akt Ser473 phosphorylation, thereby inhibiting GSK3β and FOXO1 while promoting glycolysis. This metabolic shift enhances the expression of reparative genes such as *Arg1* and *Klf4*, which negatively regulate inflammatory responses^24^. Furthermore, PIK3AP1 inhibits NLRP3 and NLRC4 inflammasome activation in macrophages^42^. Accordingly, our findings suggest that PIK3AP1 may function as a key upstream regulator of mTORC2–Akt signaling in macrophages. These findings identify the PIK3AP1–mTORC2 axis as a critical mediator of PKCδ deficiency-associated protection, highlighting its pivotal role in controlling macrophage-driven inflammation and metabolic dysfunction in atherosclerosis.

Our findings indicate that PKCδ deficiency increases fibrous cap thickness, defined as the minimum distance between the necrotic core and the lumen in hematoxylin and eosin-stained sections^43^. The thickness of the fibrous cap may have bidirectional clinical implications. In advanced lesions, persistent plaque growth or excessive matrix accumulation may lead to luminal narrowing and impaired blood flow. However, fibrous cap thickness is a primary hallmark of plaque stabilization; mechanistically, a thicker cap rich in smooth muscle cells and extracellular matrix provides the structural integrity necessary to attenuate mechanical stress at the plaque surface^44^. Specifically, thin cap fibroatheromas, defined by a fibrous cap thickness of <65 μm, are strongly associated with plaque vulnerability in humans^45,46^. We proposed that the increased fibrous cap thickness observed in *Ldlr*^*-/-*^*+PKCδ*^*-/-*^ mice indicates a more stable plaque phenotype. Moreover, *PKCδ*^*-/-*^ BMDMs exhibited increased TGFβ and reduced MMP9 secretion, suggesting a shift toward extracellular matrix preservation. Overall, these findings suggest that PKCδ deficiency promotes plaque stability, at least in part, through modulation of macrophage-mediated matrix remodeling.

We show the distinct effects of PKCδ deficiency between *Ldlr*^*-/-*^*+PKCδ*^*-/-*^ mice and BMT model mice. Global PKCδ knockout significantly reduced the lesion area, whereas hematopoietic-specific deficiency via BMT did not, suggesting cell type-specific roles in atherosclerosis. This difference is partly attributable to the systemic lipid-lowering effects observed in global knockout mice, indicating that PKCδ regulates lipid metabolism primarily through nonhematopoietic tissues. Consistent with these findings, Bezy et al. reported that hepatic *Prkcd* overexpression elevates serum cholesterol^47^. Notably, both models similarly enhanced plaque stability by reducing the necrotic core size and increasing the fibrous cap thickness, highlighting macrophage PKCδ as a key driver of plaque destabilization, consistent with its role in macrophage death-mediated necrotic core formation^48^. Collectively, these findings emphasize the distinct yet complementary roles of PKCδ in modulating lipid metabolism and plaque vulnerability in atherosclerosis.

In the present study, circulating IL-18, but not other measured inflammatory cytokines, was reduced in hematopoietic PKCδ-deficient BMT mice. Because plaque macrophages primarily produce IL-18 and require caspase 1-dependent maturation, this reduction may reflect decreased macrophage inflammasome activity rather than a generalized suppression of systemic inflammation^49,50^. Previous studies have shown that IL-18 is enriched in symptomatic and ulcerated human plaques and promotes IFNγ-mediated inflammation and matrix metalloproteinase expression, both linked to plaque destabilization^49,50^. Importantly, circulating IL-18 independently predicts cardiovascular mortality in patients with coronary artery disease^51^. Together, these findings suggest that IL-18 serves as a macrophage-specific marker linking hematopoietic PKCδ deficiency to reduced inflammasome-driven inflammation and improved plaque stability.

In our dataset, we found a positive correlation between *PRKCD* expression and canonical pyroptosis markers (*NLRP3, CASP1*, and *GSDMD*) in human atheroma. We further performed complementary functional studies and demonstrated that PKCδ deficiency in macrophages markedly suppressed the canonical NLRP3 inflammasome pathway, as indicated by reduced NLRP3 expression, caspase 1 activation, and GSDMD cleavage. Consistently, *Ldlr*^*-/-*^*+PKCδ*^*-/-*^ mice exhibited decreased expression of NLRP3, caspase 1, GSDMD, and IL-1β within atherosclerotic lesions. Our data suggest that PKCδ may modulate inflammasome activation through mtROS production. Together, these findings suggest that PKCδ plays a direct role in inflammasome activation and macrophage pyroptosis in atherosclerotic plaques.

We propose that PKCδ may function predominantly as a local regulator within the atherosclerotic plaque microenvironment rather than as a circulating biomarker. This interpretation is consistent with prior studies indicating that key mechanisms driving plaque destabilization are largely confined to the vessel wall and not necessarily reflected in systemic circulation^52,53^. These findings suggest that the lack of a detectable plasma difference does not preclude a pathogenic role for PKCδ; instead, it highlights its function as a local regulator of plaque inflammation and cellular dysfunction.

Our study reveals a novel finding that hematopoietic-specific PKCδ deficiency reduces plaque complexity in HCD-fed *Ldlr*^*-/-*^ mice, which contrasts with the results of Li et al., who reported increased plaque development in high-fat diet-fed *Apoe*^*-/-*^ mice with macrophage-specific PKCδ knockout^14^. Li et al. used *Apoe*^*-/-*^; PKCδ^flox/flox^; LyzM-Cre^+^ mice, which eliminate PKCδ in the myeloid lineage from embryonic development, potentially allowing for genetic compensation by the upregulation of redundant genes^54^. Our BMT approach, however, restricted PKCδ deficiency to adult hematopoietic-derived cells, possibly avoiding such compensatory mechanisms and revealing a more pronounced phenotype. Moreover, Rahman et al. demonstrated that Lyz2Cre expression increases insulin sensitivity while reducing macrophage phagocytic activity, effects that may independently influence atherosclerosis development^55^. In addition, differences in mouse atherogenic models may contribute to divergent outcomes. Previous studies have suggested that *Ldlr*^*-/-*^ mice develop atherosclerosis primarily in response to HCD owing to defective LDL clearance, whereas *Apoe*^*-/-*^ mice manifest profound hypercholesterolemia, monocytosis, and rapid atherogenesis even under normal dietary conditions, reflecting broader disruptions in lipid and immune homeostasis^56^. Regarding the diet, Li et al. used an atherogenic diet (TD.02028; Harland Laboratory) containing 12.5 g/kg cholesterol, whereas our HCD (D02031507) contained only 3 g/kg cholesterol (a 4.2-fold difference). In addition, TD.02028 contains 5.0 g/kg cholic acid, whereas D02031507 contains no bile acids. Cholic acid enhances cholesterol absorption by improving micelle solubilization^57^, whereas its absence reduces cholesterol absorption and decreases atherosclerosis by 50%^58^. The greater atherogenic potential of TD.02028 stems from its higher cholesterol content and increased cholesterol bioavailability. These results highlight the importance of developmental timing and model selection in studying the role of PKCδ in atherosclerosis and provide new insights into its context-dependent function in cardiovascular disease.

Our study has several limitations. First, the BMT approach introduces potential confounding from other transplanted immune cells, and murine atherosclerosis models exhibit fundamental differences from human disease in plaque morphology, rupture patterns, and atherothrombotic mechanisms. Second, our mechanistic insights derive primarily from in vitro systems that oversimplify the complex inflammatory microenvironment and dynamic cellular interactions present in human atherosclerotic lesions. Third, our focus on early-to-intermediate lesions may limit generalizability to advanced plaques with necrotic cores, calcification, and intraplaque hemorrhage. Despite these differences, our findings contribute important mechanistic information that can guide future translational research.

In conclusion, our study demonstrated that PKCδ plays a critical role in the evolution of atherosclerotic plaques by regulating macrophage inflammation and metabolic reprogramming. Macrophage-specific PKCδ deficiency shrinks the necrotic core, strengthens the fibrous cap, and reduces plaque vulnerability while suppressing inflammasome activation and pyroptosis, likely via the PIK3AP1–mTORC2–α-KG axis. These results highlight the potential of targeting PKCδ as a therapeutic strategy to mitigate the risk of ACS by stabilizing atherosclerotic plaques and reducing inflammatory responses.

## Supplementary information

Supplementary information

Uncorp WB

## Data Availability

The data, analytic methods, and study materials will be maintained by the corresponding author and made available to other researchers upon request.
