# Supplementary material for: Deficiency of protein kinase Cδ reprograms macrophage immunometabolism to suppress pyroptosis and stabilize atherosclerotic plaques
Source: Exp Mol Med. 2026 Sep 10;58(9):3141–57. doi: 10.1038/s12276-026-01842-9 (PMC13633269; doi:10.1038/s12276-026-01842-9)
Supplement: Supplementary file 1 [file 12276_2026_1842_MOESM1_ESM.pdf]

Deficiency of Protein Kinase Cδ Reprograms Macrophage Immunometabolism to Suppress Pyroptosis and Stabilize Atherosclerotic Plaques

Chih-Feng Lien [a], Hsin-Yi Chang [b, c], Shu-Han Yu [d], Rou-Ling Cho [a], Sy-Jou Chen [e], Tzu-Ting Kuo [c, f], Patrick Chun Theng Chong [d], Chih-Hung Ye [d], Feng-Yen Lin [g, h], Wan-Lin Wu [i], Shin-Hua Lin [j], Chien-Sung Tsai [k], and Chin-Sheng Lin [a,*]

[a] Division of Cardiology, Department of Internal Medicine, Tri-Service General Hospital, National Defense Medical University, Taipei, Taiwan.

[b] Graduate Institute of Medical Sciences, National Defense Medical University, Taipei, Taiwan.

[c] Department of Research and Development, National Defense Medical University, Taipei, Taiwan.

[d] Institute of Biotechnology, National Taiwan University, Taipei, Taiwan.

[e] Department of Emergency Medicine, Tri-Service General Hospital, National Defense Medical University, Taipei, Taiwan.

[f] Ph.D. Program for Cancer Molecular Biology and Drug Discovery, College of Medical Science and Technology, Taipei Medical University and Academia Sinica, Taipei, Taiwan.

[g] Department of Internal Medicine, School of Medicine, Taipei Medical University, Taipei, Taiwan.

[h] Division of Cardiology, Department of Internal Medicine, Taipei Medical University Hospital, Taipei, Taiwan.

[i] Department of Discovery Science and Technology, Gilead Sciences, California, USA.

[j] Division of Nephrology, Department of Internal Medicine, Tri-Service General Hospital, National Defense Medical University, Taipei, Taiwan.

[k] Division of Cardiovascular Surgery, Department of Surgery, Tri-Service General Hospital, National Defense Medical University, Taipei, Taiwan.

* Corresponding author

Corresponding author's contact information

Chin-Sheng Lin M.D, Ph.D.

Division of Cardiology, Tri-Service General Hospital, No. 325, Sec. 2, Cheng-Kung Rd., Neihu 114, Taipei, Taiwan. Tel: 886-2-8792-7160; FAX: 886-2-6601-2656; E-mail: littlelincs@gmail.com

SUPPLEMENTAL MATERIAL

Materials and reagents

Recombinant human IFNγ (Cat# 300-02), recombinant murine IFN-γ (Cat# 315-05), and recombinant murine IL-4 (Cat# 214-14) were obtained from PeproTech. Lipopolysaccharide (LPS, Cat# L2880) and 2-deoxy-D-glucose (2-DG, Cat# D6134) were obtained from Sigma. Nigericin (Nig, Cat# tlrl-nig) was obtained from InvivoGen. JR-AB2-011 (JR, Cat# HY-122022) and dimethyl α-ketoglutarate (DKG, Cat# HY-44134) were obtained from MedChemExpress (MCE). The following antibodies were obtained from different sources as indicated: CD68 (Cell Signaling Technology Cat# 97778, RRID:AB_2928056), p70 S6 kinase (Cell Signaling Technology Cat# 9202, RRID:AB_331676), phospho-p70 S6 kinase (Thr389) (Cell Signaling Technology Cat# 9234, RRID:AB_2269803), total Akt (Cell Signaling Technology Cat# 4691, RRID:AB_915783), phospho-Akt (Ser473) (Cell Signaling Technology Cat# 4060, RRID:AB_2315049), α-smooth muscle actin (α-SMA) (Abcam; Cat# ab5694; RRID:AB_2223021), NLRP3 (Novus; Cat# NBP2-12446; for IF; RRID: AB_2750946), IL-1β (Abcam; Cat# ab9722; RRID: AB_308765), PKCδ (cell signaling; Cat# 2058S), GSDMD (Novus; Cat# NBP2-33422; for WB; RRID: AB_2687913), GSDMD (Proteintech; Cat# 20770-1-AP; for IF; RRID: AB_10696319), Caspase-1 (Novus; Cat# NBP1-45433; for WB and IF; RRID:

AB_10008900), Caspase-1 (adipogen; Cat# AG-20B-0042; for WB; RRID: AB_2490248), MHC II (Bioss Cat# bs-8481R, RRID:AB_2935882), PIK3AP1 (Proteintech Cat# 27903-1-AP, RRID:AB_3086007), GAPDH (GeneTex; Cat# GTX100118; RRID:AB_1080976), Peroxidase AffiniPure goat anti-rabbit IgG (H+L) (Jackson ImmunoResearch Labs; Cat# 111-035-003; RRID:AB_2313567), Peroxidase AffiniPure goat anti-mouse IgG (H+L) (Jackson ImmunoResearch Labs; Cat# 115-035-003, RRID:AB_10015289), goat anti-rabbit IgG heavy and light chain antibody DyLight® 488 conjugated (Bethyl; Cat# A120-101D2; RRID:AB_10631073), and goat anti-rabbit IgG heavy and light chain antibody DyLight® 650 conjugated (Bethyl; Cat# A120-101D5; RRID:AB_10630564).

En face ORO staining

The aortae were immersed in 4% PFA for 2 days prior to staining with Oil Red O (ORO; Cat# 00625, Sigma-Aldrich, St. Louis, MO, USA). The aortae were subsequently dissected by severing the brachiocephalic artery, left common carotid artery, left subclavian artery, and a segment of the descending thoracic aorta. An incision was then made along the inner curvature of the aorta. The aortae were flattened and secured onto a black plate with pins and imaged within 3 days. Images were acquired using an iPhone XR (Apple Inc., Cupertino, CA, USA).

Histological analysis

The collected hearts were fixed in 4% paraformaldehyde (PFA) for 3 days and subsequently transferred to 75% ethanol (EtOH) for 2 days. Paraffin sections (5-μm thick) were prepared for further staining. Hematoxylin–eosin (H&E; Cat# H-3502, Vector Laboratories, Burlingame, CA, USA) staining was used to analyze the lesion area, necrotic core, and fibrous cap thickness. Picrosirius red staining (Cat# SRS250, ScyTek Laboratories, Logan, UT, USA) was performed to assess the collagen content. H&E images were captured using a light microscope (Olympus BX43, Japan) equipped with manual WSI software. Quantitative analysis was performed using ImageJ software. The formula for calculating the plaque vulnerability index was as follows: [necrotic core area (% of plaque area) + $CD68^+$ area (% of plaque area)]/[α-SMA^+ area (% of plaque area) + collagen content (% of plaque area)][1].

Immunohistochemistry and immunofluorescence analysis

Paraffin-embedded 5 μm thick sections were first deparaffinized and rehydrated. The samples were subsequently treated with 3% hydrogen peroxide (H_2O_2) for 20 minutes, heated at 97 °C for 40 minutes in a target retrieval solution (Cat# S1699, Dako, Carpinteria, CA, USA), and then cooled for 60 minutes at room temperature. The

sections were then blocked in 3% goat serum in PBS for 1 hour at room temperature and incubated overnight at 4 °C with a primary antibody diluted in Dako Real Antibody Diluent (Cat# S2022, Dako, Carpinteria, CA, USA). Following PBS washes, the sections were incubated for 10 minutes with biotinylated goat anti-polyvalent antibodies (Cat# ab64261, Abcam, Cambridge, UK) and then for another 10 minutes with streptavidin peroxidase (Cat# ab64261, Abcam, Cambridge, UK). DAB development was performed for 10 minutes at room temperature, followed by counterstaining with hematoxylin. The sections were mounted in mounting medium (Cat# 3801731, Leica Biosystems, IL, USA), and images were captured using a light microscope (Olympus BX43, Japan) with manual WSI software. ImageJ software was used for quantitative analysis.

For immunofluorescence (IF) analysis, the fixed sections were incubated in blocking solution for 10 minutes at room temperature and then with primary antibodies (diluted 1:50–1:500) overnight at 4 °C. The following day, the sections were incubated with secondary antibodies (1:500 dilution) conjugated to DyLight 488 or DyLight 650 for 1 hour at room temperature. The sections were then mounted with medium containing DAPI (Vector Laboratories, Burlingame, CA, USA). IF images were obtained using a slide scanner (Zeiss, Germany), and the colocalization area was measured using ImageJ software.

Opal multiplex immunofluorescence staining

In this study, we used an innovative multiplexed IHC imaging technique (Opal Polaris 7-Color IHC Manual Detection Kit; Akoya Biosciences®, Massachusetts, USA), which consists of a repeated stain–image–dye–inactivation sequence using direct antibody–fluorophore conjugates, and performed single-cell segmentation of atherosclerotic plaques. Tissue pretreatment and antibody incubation were performed as previously described [2], except that the secondary antibody was substituted with the Novolink Polymer Detection Systems (Leica). The slides were washed, and the designated tyramide signal amplification (TSA) dye (Opal 7-Color Kit; Akoya Biosciences®, Massachusetts, USA) was applied for 10 minutes according to the manufacturer's instructions. The slides were heated in retrieval buffer in a steamer to strip primary and secondary antibodies and then pretreated with the next staining target. The TSA dyes precipitated around the antibody complex and emitted fluorescent signals for visualization. The cooldown, blocking, antibody, and Opal dye incubation steps were then repeated for a total of four staining cycles. The primary antibodies used for immunostaining in separate incubation were as follows: MHC II (1:400), CD68 (1:200), and DAPI.

Lipid profile analysis

The plasma samples were analyzed to determine lipid profiles. Triglyceride (TG), total cholesterol (TOCH), high-density lipoprotein (HDL), glutamic oxaloacetic transaminase (GOT), and glutamic pyruvic transaminase (GPT) levels were measured using FUJI Dri-Chem slides on a Fuji Dri-Chem FDC NX500 analyzer (Fuji Photo Film, Tokyo, Japan). Low-density lipoprotein (LDL) levels were calculated using the Friedewald formula [3].

Murine plasma cytokine analysis

The secretion of cytokines, including TNF-α, IL-12, IL-6, IL-18, and IL-33 from mouse plasma was measured using the ProcartaPlex Multiplex Immunoassays (Invitrogen; Thermo Fisher Scientific, Waltham, MA, USA) according to the manufacturer's instructions.

Cell culture

Bone marrow-derived macrophages (BMDMs) were isolated from 8–12-week-old WT and $PKC\delta^{-/-}$ mice. The cells were then differentiated in DMEM supplemented with 5% fetal bovine serum, 1% penicillin/streptomycin, and 20% L929-conditioned medium for 5–7 days. To induce an M1-like inflammatory macrophage phenotype in BMDMs,

the cells were treated with 20 ng/ml LPS and 20 ng/ml IFN-γ for 24 hours in 5% serum-containing media. All cell lines were obtained from the ATCC (Manassas, VA, USA). L929 (ATCC Cat# CCL-1, RRID:CVCL_0462) cells were cultured in DMEM, and THP-1 (RRID:CVCL_0006) cells were cultured in RPMI-1640 medium supplemented with 10% fetal bovine serum. The cells were maintained in a humidified incubator at 37 °C and 5% CO_2. To differentiate THP-1 cells into macrophages, they were incubated with 100 ng/ml phorbol 12-myristate13-acetate (PMA) for 72 hours. To induce an M1-like inflammatory macrophage phenotype in THP-1 cells, the cells were treated with 10 ng/ml LPS and 10 ng/ml IFN-γ for 24 hours.

shRNA-mediated knockdown

To specifically block PKCδ, a lentivirus containing isoform-specific shRNA (from the National RNAi Core Facility, Taiwan) was produced. THP-1 cells were infected with the virus at a multiplicity of infection (MOI) of 5 in the presence of 8 μg/mL polybrene. The transfected cells were then selected with 1 μg/ml puromycin to generate stable clones.

Real-time quantitative PCR (RT–qPCR)

Total RNA was extracted from the cells using an RNeasy Mini Kit (Cat# 74104; Qiagen, USA) following the manufacturer's instructions. The extracted RNA was then reverse transcribed into cDNA using the iScript™ Reverse Transcription Supermix (Bio-Rad). For quantitative PCR (qPCR) analysis, KAPA SYBR Green was used, and the reactions were conducted on a LightCycler Real-Time PCR System (Roche). Gene expression data were normalized to the internal control gene, 18S rRNA, and presented using the comparative Ct method. The primers used for RT–qPCR amplification are listed in Supplementary Table 1.

RNA sequencing and data analysis

Total RNA from the BMDMs of WT mice and $PKC\delta^{-/-}$ mice treated with vehicle or LPS/IFNγ was extracted and purified using a RNeasy Mini Kit (Cat# 74104; Qiagen, USA). The RNA-seq service was provided by the Instrument Center of National Defense Medical University. The resulting dataset has been deposited in the Gene Expression Omnibus (GEO) under accession number GSE327092. The gene expression differences and upstream pathway predictions were calculated using CLC Genomics Workbench, Ingenuity Pathway Analysis (IPA), and Gene Set Enrichment Analysis (GSEA) software. The pathway analysis plots were generated using an R package.

Phosphoproteomic analysis

The cells were homogenized in lysis buffer (12 mM sodium deoxycholate, 12 mM sodium N-lauroylsarcosinate, 100 mM Tris-HCl, pH 9.0), supplemented with a protease inhibitor cocktail, and the protein concentration was quantified using a Pierce™ BCA Protein Assay Kit (Thermo Fisher Scientific). The extracted proteins were reduced with 10 mM TCEP (Merck), alkylated with 25 mM chloroacetamide (Merck), and digested with LysC (Wako) for 3 hours, followed by trypsin (Promega) for 16 hours in 50 mM ammonium bicarbonate (Sigma–Aldrich) at 37 °C. The digestion was terminated by the addition of 1% trifluoroacetic acid (TFA), and the detergent was extracted with ethyl acetate. The peptide mixture was desalted with reversed-phase StageTips and quantified using the Pierce™ Quantitative Colorimetric Peptide Assay (Thermo Fisher Scientific). Phosphopeptides were enriched by the HAMMOC method[4] and labeled with TMTpro 16-plex (Thermo Fisher Scientific) in 200 mM HEPES (Sigma–Aldrich) according to the manufacturer's instructions. The samples were acidified, combined, desalted, and fractionated by bRP[5] with C18 StageTip [6].

Mass spectrometry analysis was performed on an Orbitrap Fusion Lumos Tribrid mass spectrometer coupled to an Ultimate 3000 nanoLC system (Thermo Fisher Scientific). The TMT-labeled phosphopeptide samples from each fraction were dissolved in 2% ACN with 0.1% FA. Separation was performed using a C18 Acclaim

PepMap NanoLC column (Thermo Fisher Scientific) and a 2–40% ACN gradient over 90 minutes at a flow rate of 300 nL/min. The mass spectrometer was operated in data-dependent mode. The MS1 spectra in the m/z range of 350–1,700 were acquired in the orbitrap with a resolution of 120,000 at m/z 200, an AGC target of 5×10^5, and a maximum injection time of 50 ms. The instrument was set to run in top speed mode with 3-second cycles for survey and the MS/MS scans. Peptide ions with charge states ranging from 2 to 7 were selected with an isolation width of 1.4 Da for fragmentation by higher-energy collisional dissociation at a normalized collision energy of 38%. The resulting fragment spectra were acquired in the Orbitrap mass analyzer at a resolution of 60,000. An AGC target of 5×10^4 was set for MS/MS analysis, with previously selected ions dynamically excluded for 60 seconds.

Peptides were identified using MaxQuant software[7] (version 2.4.0.0) against the *Mus musculus* SwissProt Database (version 2023_05) with the Andromeda peptide search engine. The MS2 reporter ion intensities were adjusted for TMTpro 16-plex isobaric labels. Variable modifications included phosphorylation of serine, threonine, and tyrosine (STY), methionine oxidation, and N-terminal protein acetylation, whereas carbamidomethylation of cysteine was set as a fixed modification. Trypsin/P was the enzyme specificity, allowing a maximum of two missed cleavages. The “match between runs” feature was enabled with default settings. The Phospho (STY)Site.txt

output file from MaxQuant was further processed and normalized using Perseus [8] (version 2.0.11.0). Only class 1 phosphopeptides with a localization probability > 0.75 were accepted for further analysis. Kinase enrichment analysis was performed using the Kinase Library [9]. Proteomics data have been deposited in the jPOST repository (ProteomeXchange Consortium) under accession number JPST004142.

TCA cycle-related metabolite analysis

The quantification of TCA cycle metabolites was performed using ultra-performance liquid chromatography triple quadrupole mass spectrometry (UPLC-QQQ MS/MS). The cells were washed with ice-cold saline three times and homogenized in ice-cold 80% methanol. The extracts were clarified by centrifugation at 17,000 × g for 20 minutes at 4 °C followed by C18 SPE. The metabolites were separated on a Shimadzu Nexera LC-40 system consisting of an SCL-40lite system controller, an LC-40D X3 pump, a SIL-40C X3 autosampler, and a CTO-40C column oven (Shimadzu, Japan). Liquid chromatography separation was performed at 40 °C on an ACQUITY UPLC HSS T3 column (2.1 × 100 mm, 1.8 μm, Waters, USA). The mobile phase consisted of 0.1% formic acid in water (A) and 0.1% formic acid in acetonitrile (B) and was used for gradient elution as follows: 0–4 min, 0–4% B; 4.1–6 min, 80% B; and 6.1 minutes, 0% B. The sample injection volume was 5 μL, and the flow rate was 0.4 mL/min. MS

detection was performed on a Shimadzu LCMS-8045 (Shimadzu, Japan) in negative ESI mode. The interface temperature was set at 300 °C, the DL temperature was 250 °C, the nebulizing gas flow rate was 2 L/min, the heating gas flow rate was 10 L/min with the heat block set at 400 °C, and the drying gas flow rate was 10 L/min. The metabolites of interest were targeted with specific MRM transitions: m/z 115 > 71 for fumaric acid, m/z 133 > 115 for malic acid, m/z 131 > 87 for oxaloacetic acid, m/z 145 > 101 for α-ketoglutaric acid, and m/z 191 > 111 for citric acid. Quantification was performed using calibration curves generated from standard solutions ranging from 7.8125 to 2,000 ng/ml. Quality-control samples were included to ensure consistent retention times and peak shapes across all runs, enabling reliable and reproducible quantification of TCA cycle metabolites in various biological samples. Data were normalized to the WT vehicle group and are presented as relative abundance.

Metabolism analysis

The glycolytic and mitochondrial activities of the macrophages were measured using a Seahorse XFp analyzer (Seahorse Bioscience). BMDMs were seeded at a density of 2.5 $\times 10^4$ cells/well in 100 μl of DMEM and allowed to adhere for 24 hours. The medium was then replaced with 200 μl of fresh DMEM with or without LPS/IFN-γ, and the cells were incubated overnight. For oxygen consumption rate (OCR) measurement, the cells

were washed twice with PBS on the following day and incubated with 180 μl of Seahorse XF DMEM base medium (Agilent; Cat# 103575-100, Santa Clara, CA, USA) containing 10 mM glucose, 1 mM sodium pyruvate (Corning; Cat# 25-000-CI, NY, USA), and 2 mM L-glutamine (Corning; Cat# 25-005-CI, NY, USA) for 1 hour in a CO_2-free incubator. The OCR was then measured following the sequential injection of 1 μM oligomycin, 1.5 μM carbonyl cyanide-4-(trifluoromethoxy)phenylhydrazone (FCCP), and 0.5 μM rotenone plus 0.5 μM antimycin A. To measure the extracellular acidification rate (ECAR), the cells were washed twice with PBS on the following day and incubated with 180 μl of Seahorse XF DMEM base medium containing 1 mM sodium pyruvate and 2 mM L-glutamine for 30 minutes in a CO_2-free incubator. The ECAR was then measured following the sequential injection of 10 mM glucose, 1 μM oligomycin, and 75 mM 2-DG. All metabolic data were normalized to the total cellular protein concentration.

Western blotting

Proteins were extracted from either cells or tissues using lysis buffer, after which the protein concentration was quantified. Equal amounts of total cellular extracts were loaded onto 10-12% sodium dodecyl sulfate–polyacrylamide gels for electrophoresis. The separated proteins were subsequently transferred onto a nitrocellulose membrane,

which was then blocked for 1 hour with 5% BSA in TBST buffer (20 mM Tris, 150 mM NaCl, and 0.05% Tween-20). The membrane was subsequently incubated overnight at 4 °C with primary antibodies (diluted 1:1,000), washed with TBST buffer, and incubated for 1 hour with secondary antibodies (diluted 1:5,000) in blocking buffer. Finally, the membrane was thoroughly washed, immersed in substrate, and visualized using a luminescent image analyzer (LAS-4000; Fujifilm, Tokyo, Japan), with quantification performed using ImageJ software.

Scanning electron microscopy (SEM)

The cells were cultured on glass coverslips and fixed in 3% glutaraldehyde in PBS for 1 hour. The fixed cells were washed with PBS and post-fixed for 30 minutes in 1% osmium tetroxide (OsO_4). The samples were then dehydrated through an ethanol series and dried at the critical point. The glass coverslips with cells were subsequently mounted on SEM stubs using adhesive carbon tape. A thin layer of gold was sputter-coated onto the sample surface to enhance conductivity. Imaging was performed using a scanning electron microscope (Apreo 2; Thermo Fisher Scientific) with secondary electron detection at an acceleration voltage of 15 kV.

Isocitrate dehydrogenase (IDH) activity assay

IDH activity was measured using a colorimetric assay kit (Abcam; Cat# ab102528) according to the manufacturer's instructions. Briefly, BMDMs ($2.5x10^5$ cells) were lysed in 200 µl of IDH assay buffer. The lysates were centrifuged at 13,000 × g for 10 minutes. The cleared supernatant was collected and diluted. NAD^+ and β-NADP were used in the reaction mixture for the IDH assay. The mixture was incubated for 3 minutes at 37 °C, followed by measurement of the OD at 450 nm using a Synergy HT Reader (BioTek Instruments). The samples were subsequently incubated for an additional 30 minutes at 37 °C, after which the OD at 450 nm was measured again.

α-Ketoglutarate assay

The α-KG concentration was measured using a colorimetric assay kit (Arigo; Cat# ARG82662) according to the manufacturer's instructions. Briefly, BMDMs ($2.5x10^5$ cells) were lysed in 200 µl of assay buffer. The lysates were centrifuged at 12,000 × g for 10 minutes at 4 °C, and the cleared supernatant was collected. The working solution was added to the samples, and the mixture was incubated for 120 minutes at 37 °C. The optical density was then measured at 540 nm using a Synergy HT Reader (BioTek Instruments). Data were normalized to the WT vehicle group and are presented as relative abundance.

Enzyme-linked immunosorbent assay (ELISA)

TGF-β1 (Cat# ELK1186MS; ELK Biotechnology, Sugar Land, TX, USA), PDGF-B (Cat# ELK11401; ELK Biotechnology, Sugar Land, TX, USA), and MMP-9 (Cat# ELK10239MS; ELK Biotechnology, Sugar Land, TX, USA) levels in BMDM culture supernatants were quantified using ELISA kits according to the manufacturers' instructions. Briefly, samples were added to the wells and incubated for the indicated time. Plates were then washed, followed by incubation with detection antibodies and streptavidin-HRP conjugate. After additional washing steps, substrate solution was added for color development in the dark, and the reaction was terminated with stop solution. Absorbance was measured at 450 nm using a SpectraMax iD5 Microplate Reader (Molecular Devices, USA).

Mitochondrial ROS assay

Mitochondrial reactive oxygen species (mtROS) production in BMDMs was assessed using MitoSOX Red mitochondrial superoxide indicator (Invitrogen; Cat# M36008, USA). Following the indicated treatments, cells were incubated with 5 μM MitoSOX for 30 min at 37°C in the dark. Cells were then washed twice with PBS, resuspended in PBS, and subjected to flow cytometric analysis using a BD FACSCalibur (BD Biosciences). Data were analyzed using FlowJo software (version 10, Tree Star Inc.).

Co-immunoprecipitation

Cell lysates were prepared using lysis buffer, and protein concentrations were determined before immunoprecipitation to ensure equal input. Protein G magnetic beads (Cytiva; Cat# 28967070) were washed with binding buffer (Cytiva; Cat# 28903059) and incubated with primary antibody for 1 hour at room temperature. The antibody-conjugated beads were then incubated with cell lysates for 1 hour at room temperature with gentle rotation. After incubation, beads were washed twice with binding buffer. Bound proteins were eluted with the elution buffer and immediately neutralized. Eluted samples were analyzed by immunoblotting.

Computational protein interaction prediction

To evaluate the potential direct interaction between PKCδ and NLRP3, in silico protein-protein interaction analyses were performed using AlphaFold Multimer (https://alphafoldserver.com) [10] and PEPPI (https://aideepmed.com/PEPPI/) [11]. For AlphaFold Multimer analysis, the amino acid sequences of PKCδ and NLRP3 were submitted to the server, and predicted complex structures were generated with corresponding confidence metrics, including interface predicted TM-score (ipTM) and

predicted TM-score (pTM). In parallel, the same protein sequences were analyzed using the PEPPI prediction platform, which estimates the likelihood of protein-protein interaction based on structural and sequence features, and outputs a log likelihood ratio (log(LR)) score. Default parameters were used for both platforms unless otherwise specified.

Gene Expression Omnibus (GEO) database analysis

The bulk mRNA array data were obtained from the Gene Expression Omnibus (GEO) datasets GSE43292 and GSE41571. The GSE43292 dataset consists of 32 paired samples of early and advanced carotid atherosclerotic plaques. Each pair includes a sample from the patient's advanced atherosclerotic plaque (classified as stage IV or higher according to the Stary classification) and a sample of macroscopically intact tissue (classified as stages I–II) [12]. The GSE41571 dataset comprises 11 samples obtained from laser microdissected, macrophage-rich regions of 5 ruptured and 6 stable human atherosclerotic plaques [13].

Single-cell RNA sequencing (scRNA-seq) analysis

scRNA-seq datasets of human atherosclerotic plaques were downloaded from the GEO database (GSE131778)[14] and reanalyzed using Cell Ranger v7.2 software (10x

Genomics). The GRCh38-2020-A reference genome was used for alignment, and the individual datasets were then aggregated using the Cell Ranger aggr function. Cells with more than 500 UMIs were selected for analysis. The raw gene expression matrix was transformed into a Loupe browser (v7.0.1). The cell clusters were manually assigned to the major cell types in accordance with established markers [14,15], and visualized using a UMAP model.

Human blood sample collection

The protocol for collecting human tissue samples was approved by the Institutional Review Board of Tri-Service General Hospital, Taipei, Taiwan (TSGHIRB No. C202105147). All experiments involving human blood samples complied with relevant guidelines, regulations, and the 1964 Helsinki Declaration. The study included patients diagnosed with stable angina (n = 52) or acute coronary syndrome (n = 55) according to the American Heart Association (AHA) guidelines. All participants underwent coronary angiography, with or without percutaneous coronary intervention, and provided written informed consent. Patient information was de-identified to ensure privacy. Blood was collected in EDTA-coated tubes, mixed several times, and centrifuged for 20 minutes at 2,000 rpm, after which the supernatant was collected.

Human plasma PKCδ analysis

Human plasma PKCδ levels were measured using an ELISA kit (FineTest; Cat# EH3588) following the manufacturer's instructions. Prepared plasma samples were applied to a 96-well plate pre-coated with the capture antibody. Following a 90-minute incubation at 37 °C, the wells were washed and then incubated with a biotin-conjugated anti-PKCδ detection antibody for 60 minutes. After additional washing, HRP-streptavidin was added, and the mixture was incubated for 30 minutes. The TMB substrate was subsequently introduced, and color development was allowed to proceed for 20 minutes in the dark. The reaction was stopped by the addition of a stop solution, and the PKCδ concentration was determined by measuring the OD at 450 nm using a SpectraMax Plus 384 Microplate Reader (Molecular Devices, San Jose, CA, USA). The concentration of PKCδ in the plasma samples was quantified by generating a standard curve from known concentrations.

SUPPLEMENTARY FIGURES

Supplementary Fig. 1

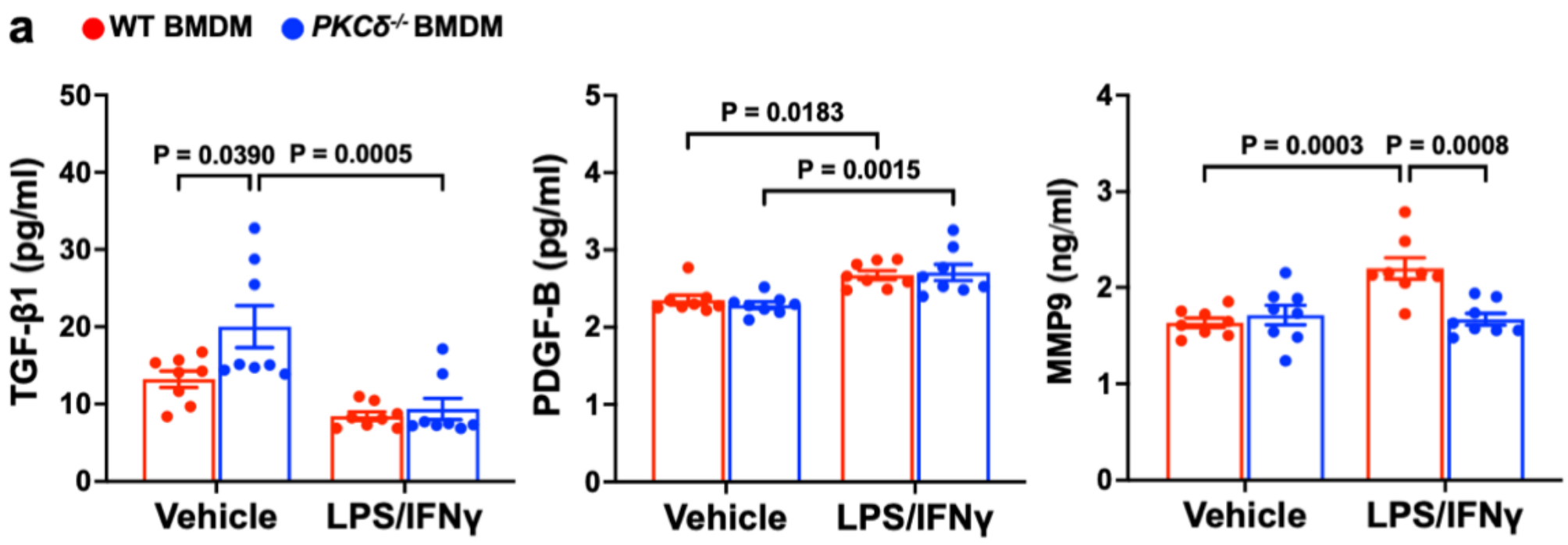

Supplementary Fig. 1. PKCδ deficiency alters TGF-β1 and MMP-9 expression in BMDMs.

a, The protein expression levels of TGF-β1, BDGF-B, and MMP9 in BMDM culture supernatants were quantified by ELISA following stimulation with LPS (20 ng/ml) and IFNγ (20 ng/ml) for 24 hours (n = 8 in each group). The data are expressed as the means ± SEMs. The *P* value was determined by two-way ANOVA with Tukey's test.

Supplementary Fig. 2

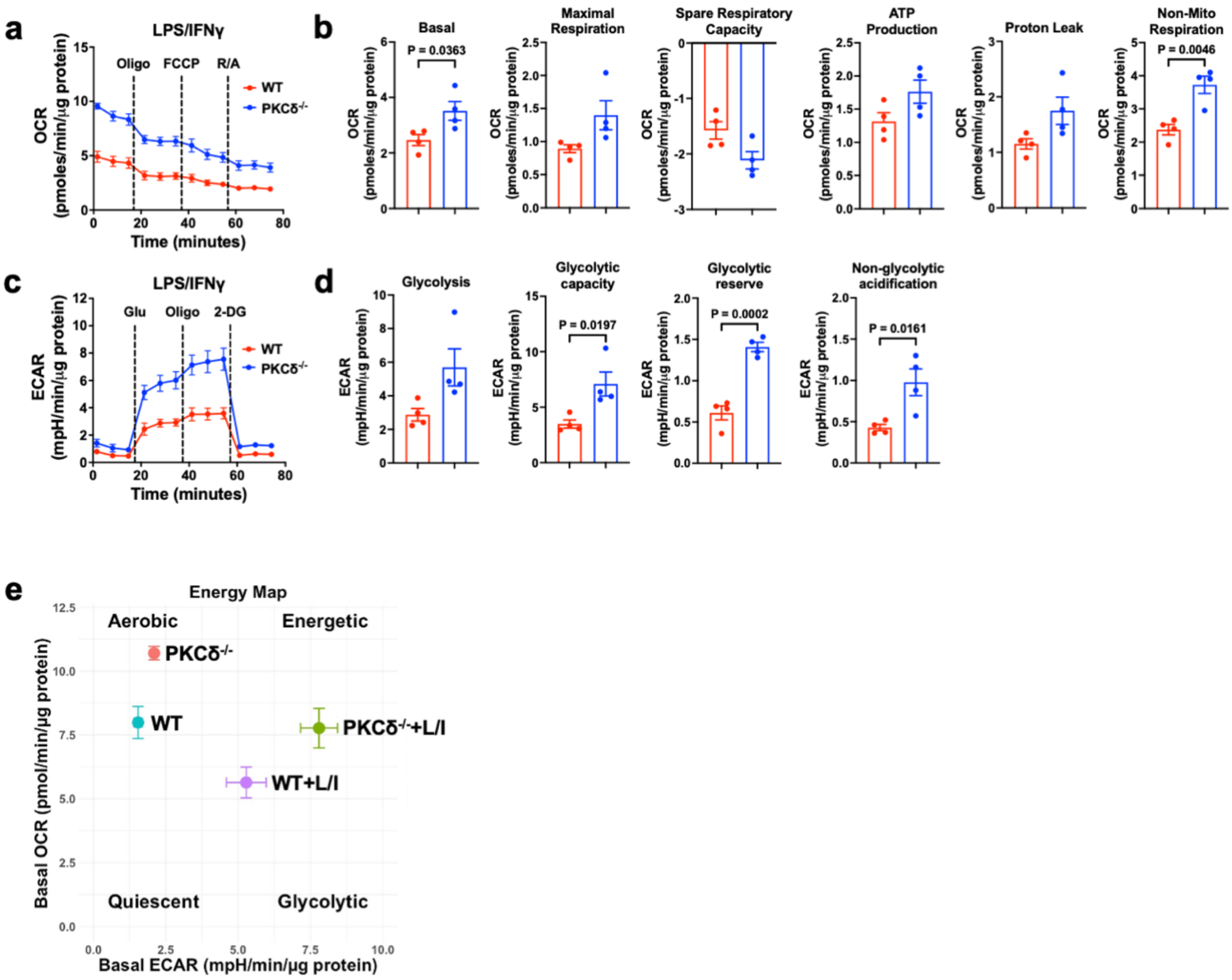

Supplementary Fig. 2. PKCδ deficiency induces changes in glycolysis and shifts the energy status of BMDMs.

a, Extracellular acidification rate (ECAR) of $PKC\delta^{-/-}$ and WT BMDMs was measured using a Seahorse analyzer. **b**, Quantification of the glycolysis parameters derived from the ECAR values (n = 4 in each group). The data are expressed as the means ± SEMs. *P* values were determined by unpaired two-tailed Student's t tests. **c**, ECAR of $PKC\delta^{-/-}$ and WT BMDMs treated with LPS/IFNγ for 24 hours, as measured using a Seahorse

analyzer. **d**, Quantification of the glycolysis parameters derived from the ECAR values (n = 4 in each group). The data are expressed as the means ± SEMs. *P* values were determined by unpaired two-tailed Student’s t tests. **e**, Energy map showing the basal OCR versus the basal ECAR in $PKC\delta^{-/-}$ and WT BMDMs treated with vehicle or LPS/IFNγ for 24 hours.

Supplementary Fig. 3

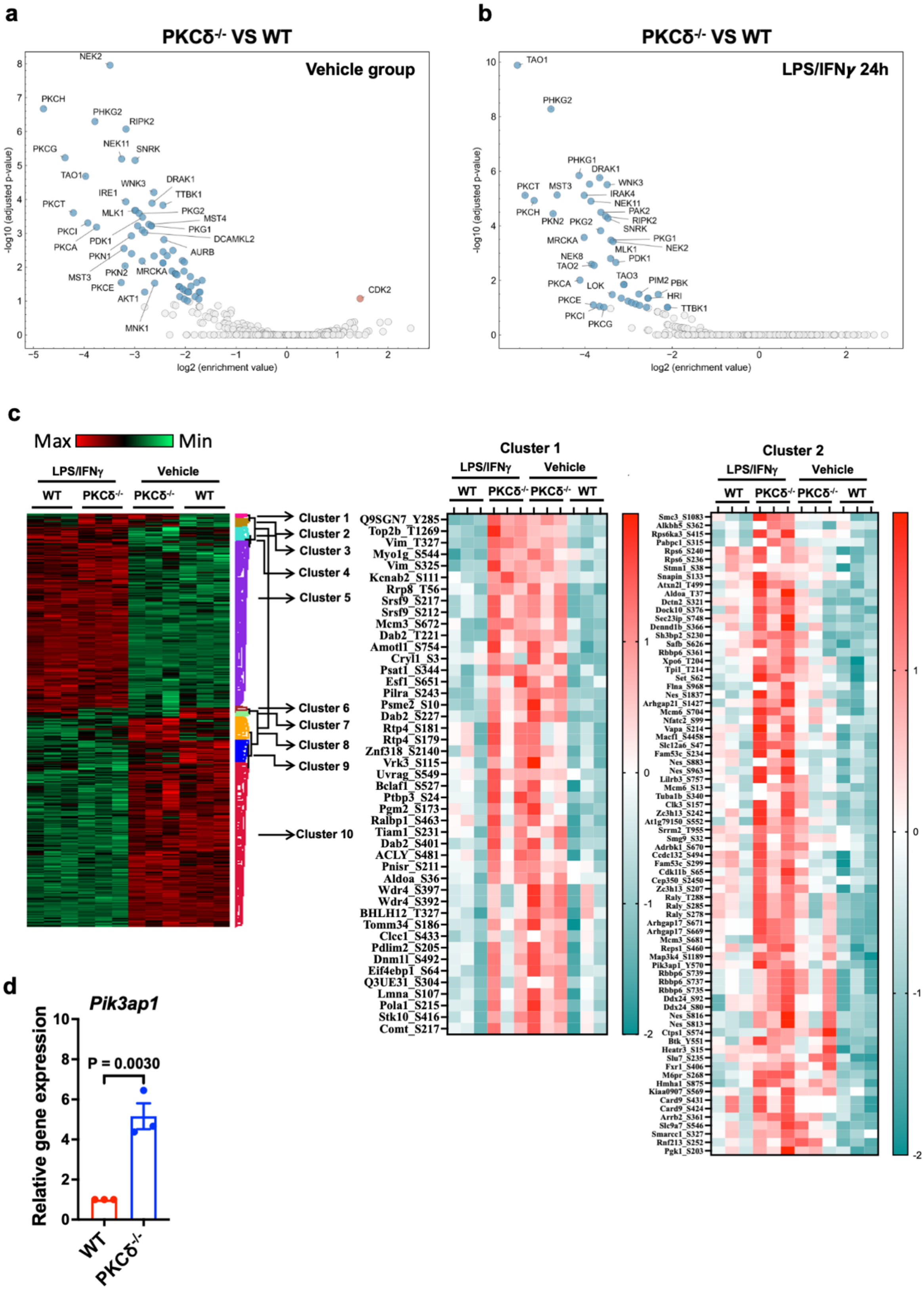

Supplementary Fig. 3. Kinase activity profiling and phosphoproteomic clustering in PKCδ$^{-/-}$ BMDMs

a and b, Kinase–substrate enrichment analysis (KSEA) was used to predict differences in kinase activity between *PKCδ*$^{-/-}$ and WT BMDMs under vehicle or LPS/INFγ stimulation (n = 3 in each group). **c**, Heatmap showing significant differences in phosphorylation sites between WT and *PKCδ*$^{-/-}$ BMDMs, with or without LPS/IFNγ stimulation. On the basis of the expression pattern, 10 clusters were identified. Clusters 1 and 2 showed an increased abundance of phosphorylation sites in *PKCδ*$^{-/-}$ BMDMs. Phosphorylation sites with increased intensities are marked in red, whereas those with decreased intensities are marked in green. **d**, The gene expression level of *Pik3ap1* was measured in BMDMs isolated from WT or *PKCδ*$^{-/-}$ mice (n = 3 in each group). The data are expressed as the means ± SEMs. *P* values were determined by unpaired two-tailed Student’s t tests.

Supplementary Fig. 4

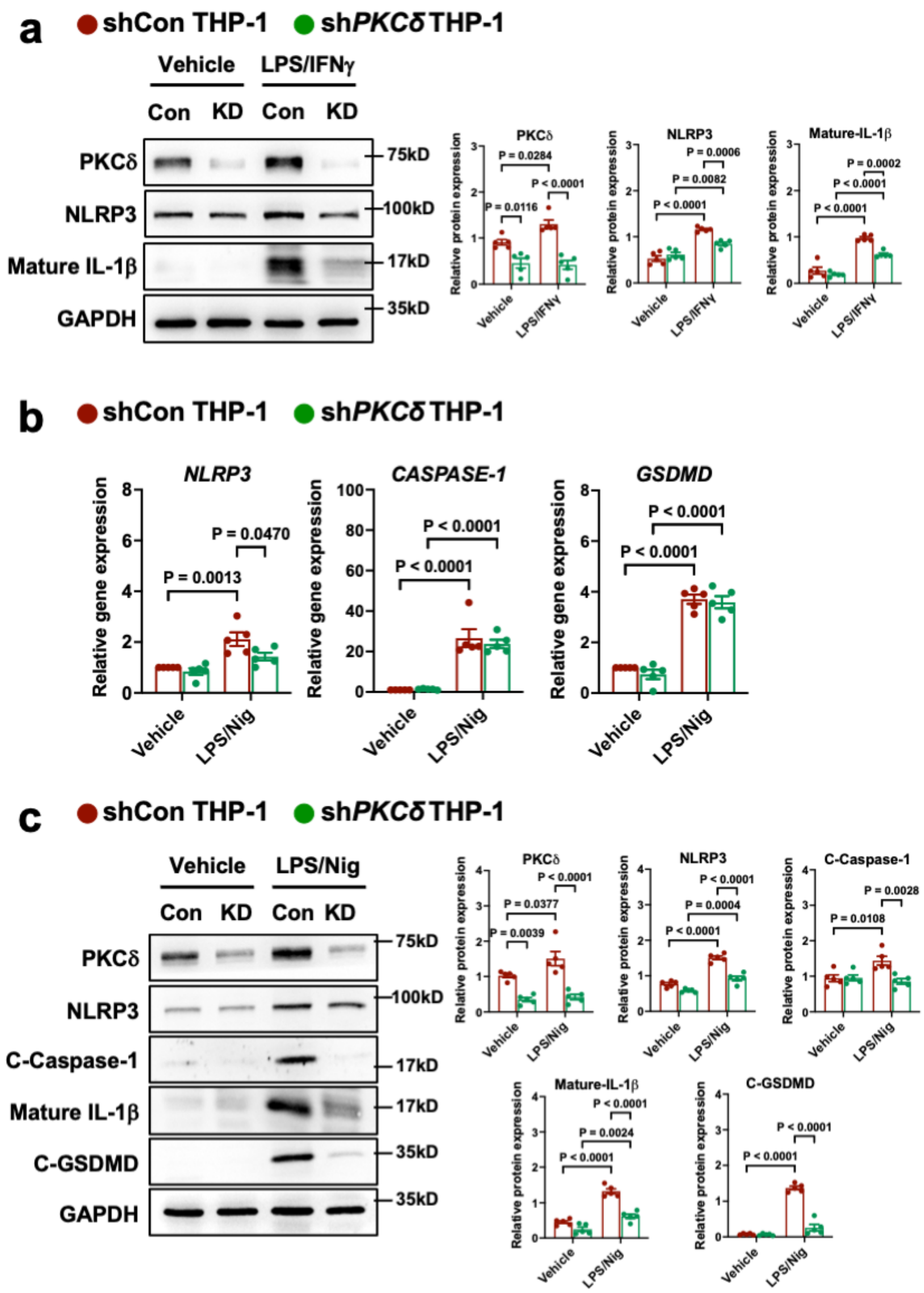

Supplementary Fig. 4. PKCδ deficiency reduces NLRP3 inflammasome-mediated pyroptosis in THP-1 cells.

a, The protein expression levels of PKCδ, NLRP3, and IL-1β were measured in THP-1 cells subjected to shRNA-mediated knockdown of PKCδ following stimulation with LPS (10 ng/ml) and IFNγ (10 ng/ml) to induce the inflammasome (n = 5 in each group). **b**, The gene expression levels of *NLRP3*, *CASP-1,* and *GSDMD* were assessed in THP-1 cells subjected to shRNA-mediated knockdown of PKCδ, followed by stimulation with LPS (100 ng/ml) for 3 hours and Nig (5 μM) for 1 hour to induce pyroptosis (n = 5 in each group). **c**, The protein expression levels of PKCδ, NLRP3, cleaved caspase-1, mature IL-1β, and cleaved GSDMD were assessed in THP-1 cells subjected to shRNA-mediated knockdown of PKCδ following stimulation with LPS (100 ng/ml) for 3 hours and Nig (5 μM) for 1 hour to induce pyroptosis (n = 5 in each group). The data are expressed as the means ± SEMs. The *P* value was determined by two-way ANOVA with Tukey's test.

Supplementary Fig. 5

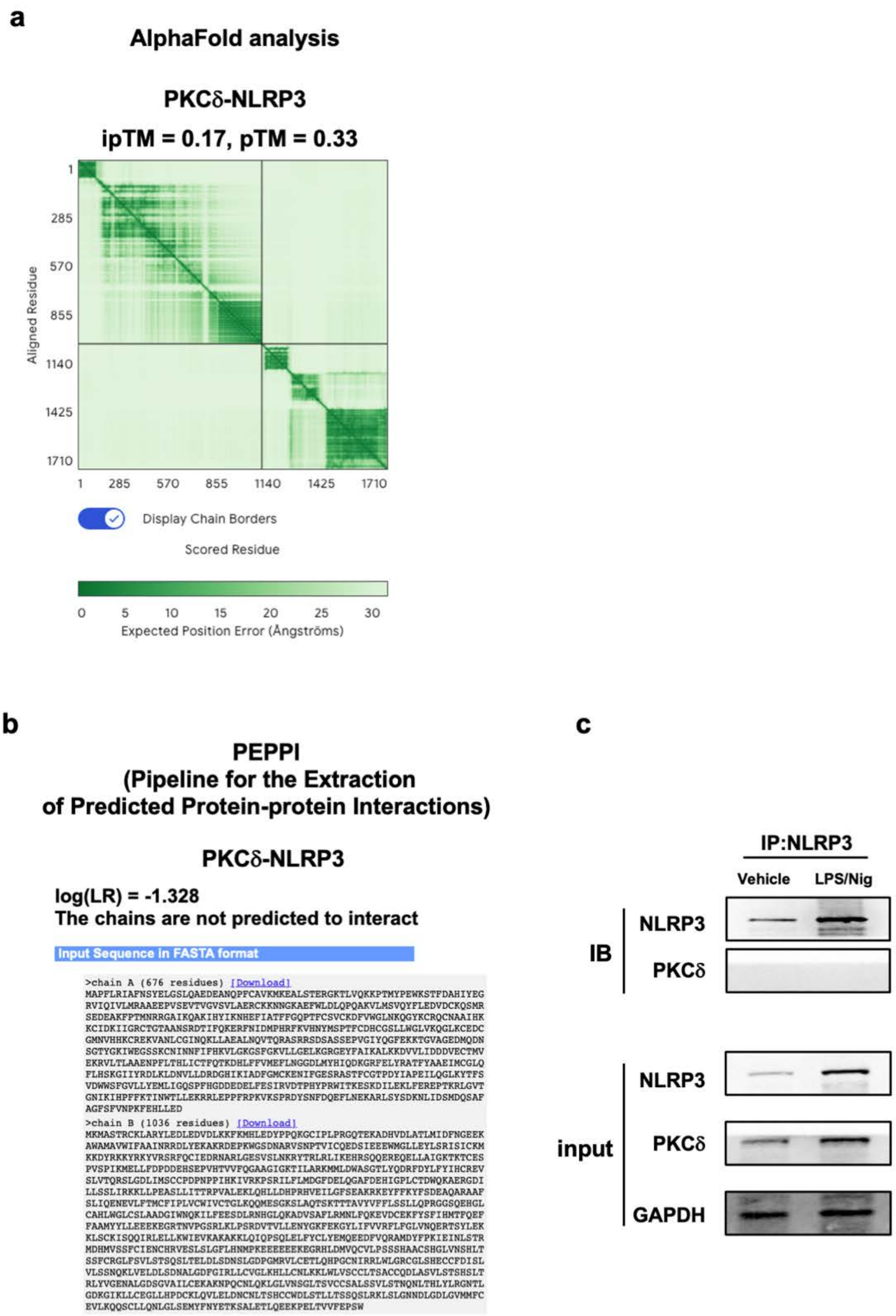

Supplementary Fig. 5. PKCδ does not directly interact with NLRP3 as predicted by computational modeling and biochemical analysis.

a, AlphaFold-Multimer analysis of the PKCδ-NLRP3 complex. The predicted aligned error (PAE) plot indicates low interface confidence, with ipTM = 0.17 and pTM = 0.33, suggesting no stable interaction. **b,** PEPPI analysis predicts a low likelihood of interaction between PKCδ and NLRP3 (log(LR) = -1.328). **c,** Co-immunoprecipitation analysis of PKCδ and NLRP3 in BMDMs treated with vehicle or LPS (100 ng/ml) for 3 hours and Nig (1 μM) for 1 hour. Cell lysates were subjected to immunoprecipitation using an anti-NLRP3 antibody (IP: NLRP3), followed by immunoblotting (IB) for PKCδ and NLRP3. No interaction between PKCδ and NLRP3 was detected under either condition. Input represents whole-cell lysates prior to immunoprecipitation, confirming comparable protein expression levels. GAPDH was used as a loading control.

Supplementary Fig. 6

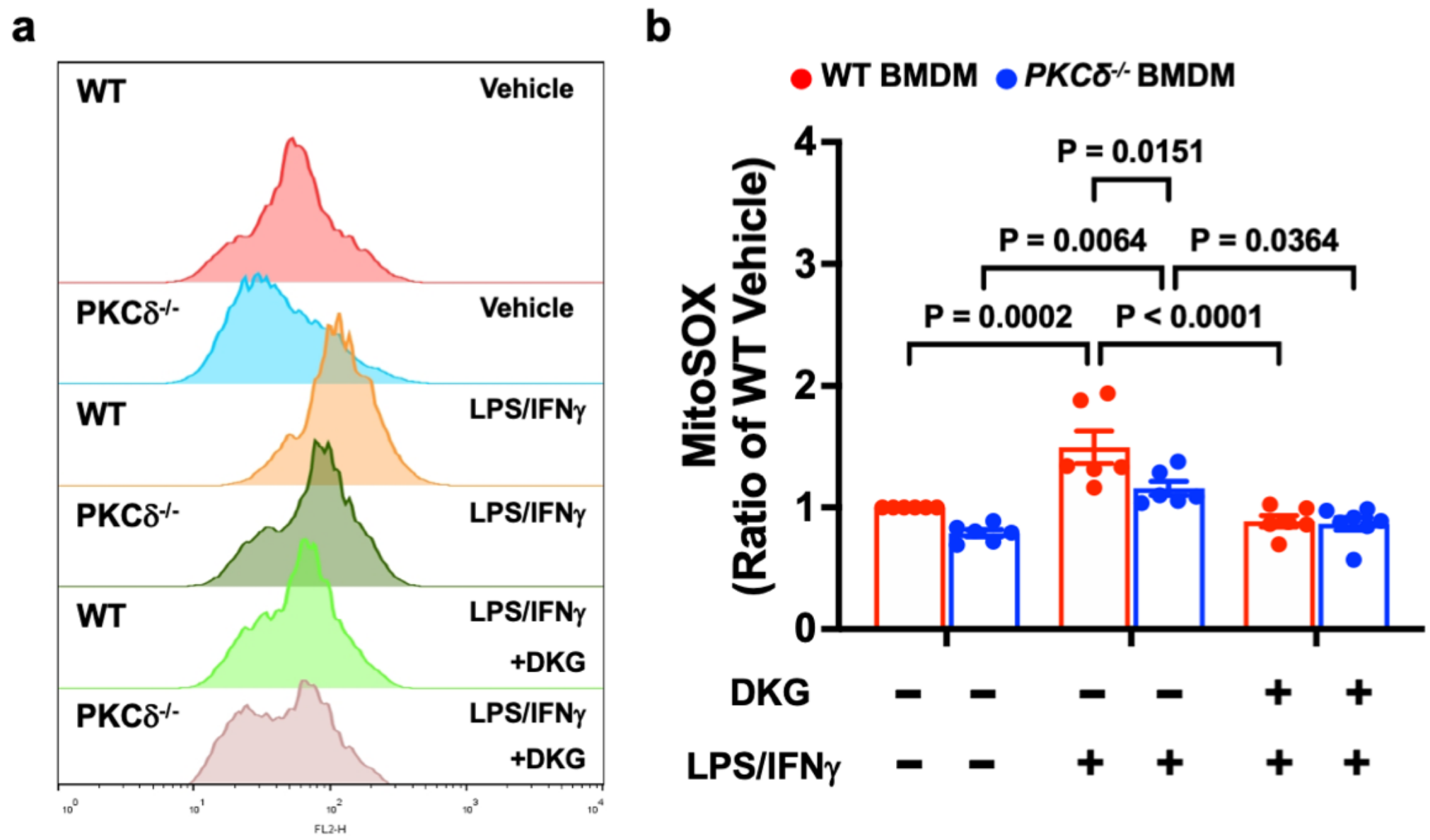

Supplementary Fig. 6. PKCδ deficiency reduces mitochondrial ROS production in BMDMs.

a, Representative flow cytometry histograms of mitochondrial ROS in WT and *PKCδ*$^{-/-}$ BMDMs under the indicated conditions, measured by MitoSOX staining. Cells were treated with vehicle, LPS (20 ng/ml), and IFNγ (20 ng/ml) for 24 hours, or LPS/IFNγ in the presence of dimethyl α-ketoglutarate (DKG). **b**, Quantification of MitoSOX fluorescence intensity, expressed as the ratio relative to WT vehicle-treated BMDMs (n = 6 in each group). The data are expressed as the means ± SEMs. The *P* value was determined by two-way ANOVA with Tukey's test.

Supplementary Fig. 7

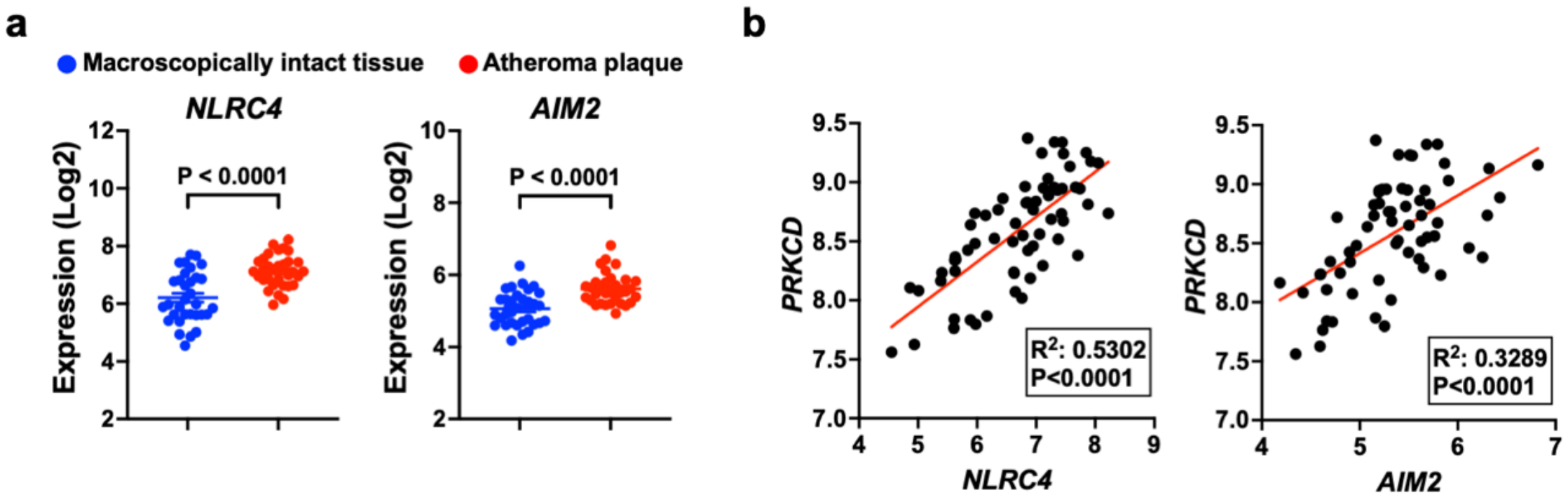

c

Groups	Pearson correlation coefficient (r)
PKC delta vs NLRP3	0.808
PKC delta vs NLRC4	0.728
PKC delta vs AIM2	0.573

Supplementary Fig. 7. Inflammasome regulator gene expression in human atheromas.

a, Gene expression analysis of *NLRC4* and *AIM2* in human atheromas compared with macroscopically intact tissue from the GSE43292 dataset (n = 32 in each group). The data are expressed as the means ± SEMs. *P* values were determined by unpaired two-tailed Student's t tests. **b**, Simple linear regression analysis between *PRKCD* expression and *NLRC4* or *AIM2* expression in human atheromas compared with macroscopically intact tissue from the GSE43292 dataset, n = 64. **c**, Pearson's correlations between the gene expression of *PRKCD* and that of *NLRP3*, *NLRC4*, or *AIM2* were analyzed in human atheromas compared with macroscopically intact tissue from the GSE43292 dataset.

Supplementary Fig. 8

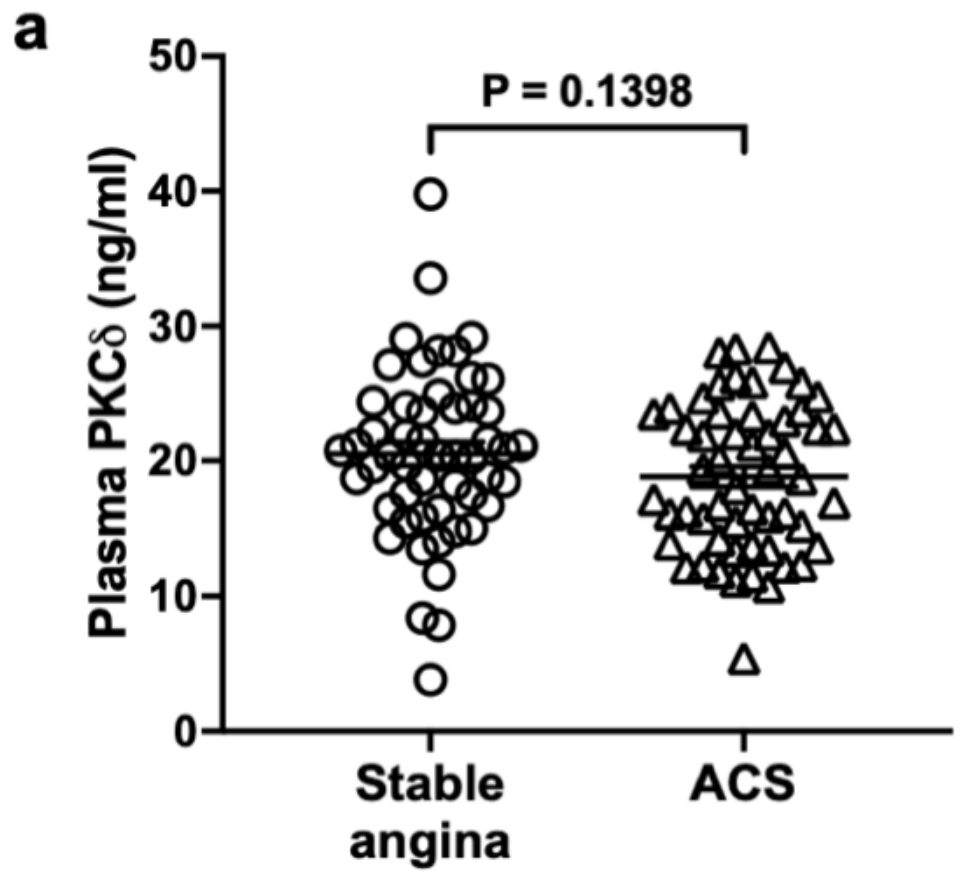

Supplementary Fig. 8. The intracoronary plasma level of PKCδ in patients

a, Plasma levels of PKCδ in patients with stable angina (n = 52) and acute coronary syndrome (ACS; n = 55) were determined using ELISA. The data are expressed as the means ± SEMs. *P* values were determined by unpaired two-tailed Student's t tests.

Supplementary Table 1. Information on qRT-PCR primers used in this study

Mouse	Gene	Forward Sequence (5'to3')	Reverse Sequence (5'to3')
1	*Tnf-α*	GAA CTT CGG GGT GAT CGG T	CTC CTC CAC TTG GTG GTT TG
2	*Il-6*	CTC TGC AAG AGA CTT CCA TCC	TGA AGT CTC CTC TCC GGA CT
3	*Il-1β*	TGC CAC CTT TTG ACA GTG ATG	ATG TGC TGC TGC GAG ATT TG
4	*Nos2*	CAA TGG CAA CAT CAG GTC GG	CGT ACC GGA TGA GCT GTG AA
5	*Nlrp3*	ATG CTG CTT CGA CAT CTC CT	AAC CAA TGC GAG ATC CTG AC
6	*Caspase1*	CAC AGC TCT GGA GAT GGT GA	TCT TTC AAG CTT GGG CAC TT
7	*Gsdmd*	TGC GTG TGA CTC AGA AGA CC	ATA AAG CTC CAG GCA GCG TA
Human	Gene	Forward Sequence (5'to3')	Reverse Sequence (5'to3')
1	*TNF-α*	CCC ATG TTG TAG CAA ACC CTC	TAT CTC TCA GCT CCA CGC CA
2	*IL-6*	TCAATATTAGAGTCTCAACCCCCA	GAA GGG CTT GTG GAG AAG G
3	*IL-1β*	CAC GAT GCA CCT GTA CGAT CA	GTT GCT CCA TAT CCT GTC CCT
4	*NLRP3*	GCA AAA AGA GAT GAG CCG AAG T	GCT GTC TTC CTG GCA TAT CAC A
5	*CASPASE1*	CCA GGA CAT TAA AAT AAG GAA ACT GT	CCA AAA ACC TTT ACA GAA GAA TCT C
6	*GSDMD*	ATG AGG TGC CTC CAC AAC TTC C	CCA GTT CCT TGG AGA TGG TCT C

Reference

1 Viola, J. R. *et al.* Resolving Lipid Mediators Maresin 1 and Resolvin D2 Prevent Atheroprogression in Mice. *Circ Res* **119**, 1030-1038 (2016).

2 Yu, S. H. *et al.* A role for paracrine interleukin-6 signaling in the tumor microenvironment in prostate tumor growth. *Prostate* **79**, 215-222 (2019).

3 Friedewald, W. T. & Halperin, M. Clofibrate in ischemic heart disease. *Ann Intern Med* **76**, 821-823 (1972).

4 Kyono, Y., Sugiyama, N., Imami, K., Tomita, M. & Ishihama, Y. Successive and selective release of phosphorylated peptides captured by hydroxy acid-modified metal oxide chromatography. *J Proteome Res* **7**, 4585-4593 (2008).

5 Batth, T. S. & Olsen, J. V. Offline High pH Reversed-Phase Peptide Fractionation for Deep Phosphoproteome Coverage. *Methods Mol Biol* **1355**, 179-192 (2016).

6 Rappsilber, J., Mann, M. & Ishihama, Y. Protocol for micro-purification, enrichment, pre-fractionation and storage of peptides for proteomics using StageTips. *Nat Protoc* **2**, 1896-1906 (2007).

7 Tyanova, S., Temu, T. & Cox, J. The MaxQuant computational platform for mass spectrometry-based shotgun proteomics. *Nat Protoc* **11**, 2301-2319 (2016).

8 Tyanova, S. & Cox, J. Perseus: A Bioinformatics Platform for Integrative Analysis of Proteomics Data in Cancer Research. *Methods Mol Biol* **1711**, 133-148 (2018).

9 Yaron-Barir, T. M. *et al.* The intrinsic substrate specificity of the human tyrosine kinome. *Nature* **629**, 1174-1181 (2024).

10 Abramson, J. *et al.* Accurate structure prediction of biomolecular interactions with AlphaFold 3. *Nature* **630**, 493-500 (2024).

11 Bell, E. W., Schwartz, J. H., Freddolino, L. & Zhang, Y. PEPPI: Whole-proteome Protein-protein Interaction Prediction through Structure and Sequence Similarity, Functional Association, and Machine Learning. *J Mol Biol* **434**, 167530 (2022).

12 Ayari, H. & Bricca, G. Identification of two genes potentially associated in iron-heme homeostasis in human carotid plaque using microarray analysis. *J Biosci* **38**, 311-315 (2013).

13 Lee, K. *et al.* Increased expression of fatty acid binding protein 4 and leptin in resident macrophages characterises atherosclerotic plaque rupture. *Atherosclerosis* **226**, 74-81 (2013).

14 Wirka, R. C. *et al.* Atheroprotective roles of smooth muscle cell phenotypic modulation and the TCF21 disease gene as revealed by single-cell analysis. *Nat Med* **25**, 1280-1289 (2019).

15 Alsaigh, T., Evans, D., Frankel, D. & Torkamani, A. Decoding the transcriptome of calcified atherosclerotic plaque at single-cell resolution. *Commun Biol* **5**, 1084 (2022).
