# Supplementary material for: Deficiency of protein kinase Cδ reprograms macrophage immunometabolism to suppress pyroptosis and stabilize atherosclerotic plaques
Source: Exp Mol Med. 2026 Sep 10;58(9):3141–57. doi: 10.1038/s12276-026-01842-9 (PMC13633269; doi:10.1038/s12276-026-01842-9)

Figure 5

Figure 5I

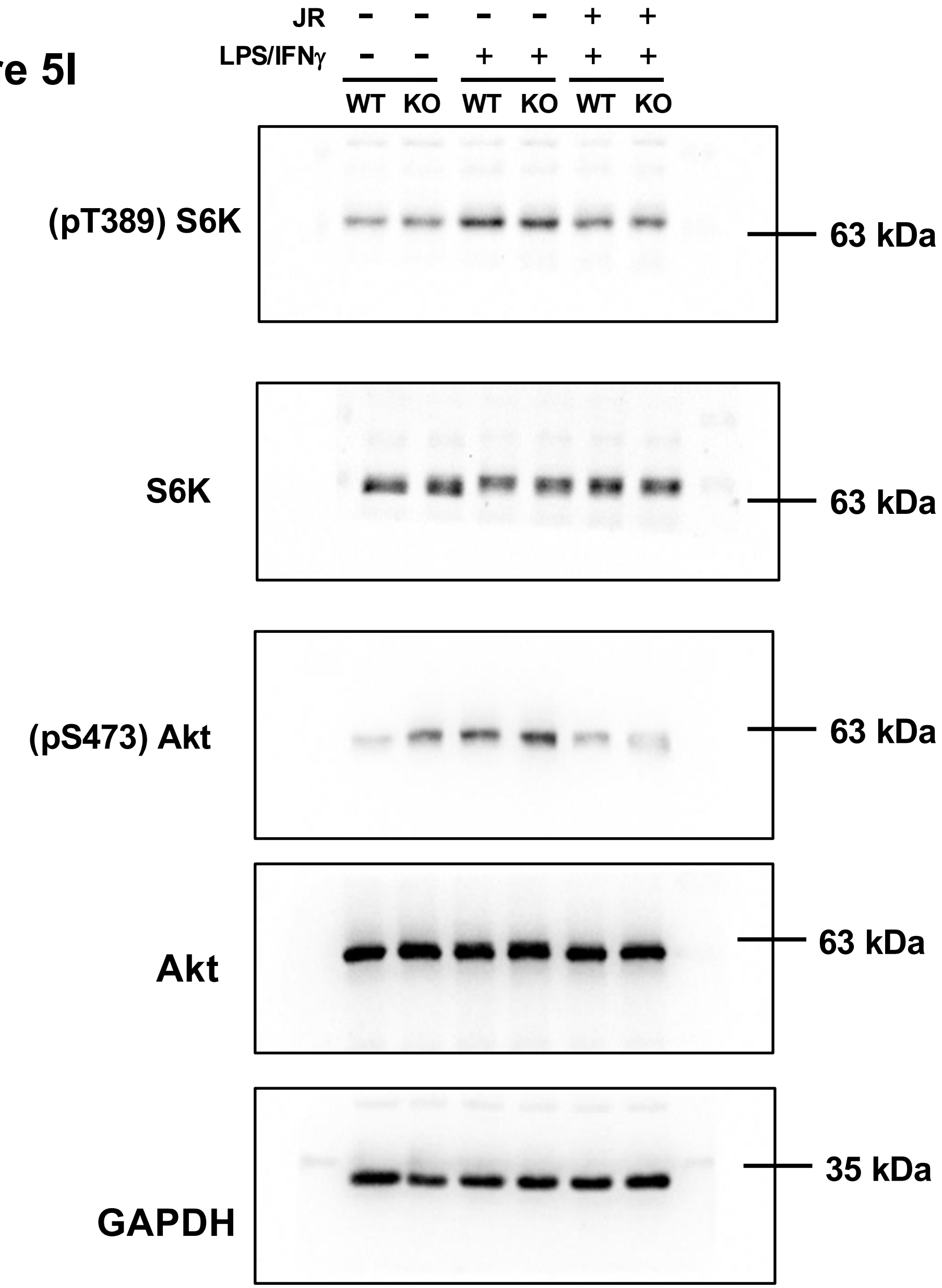

Figure 5J

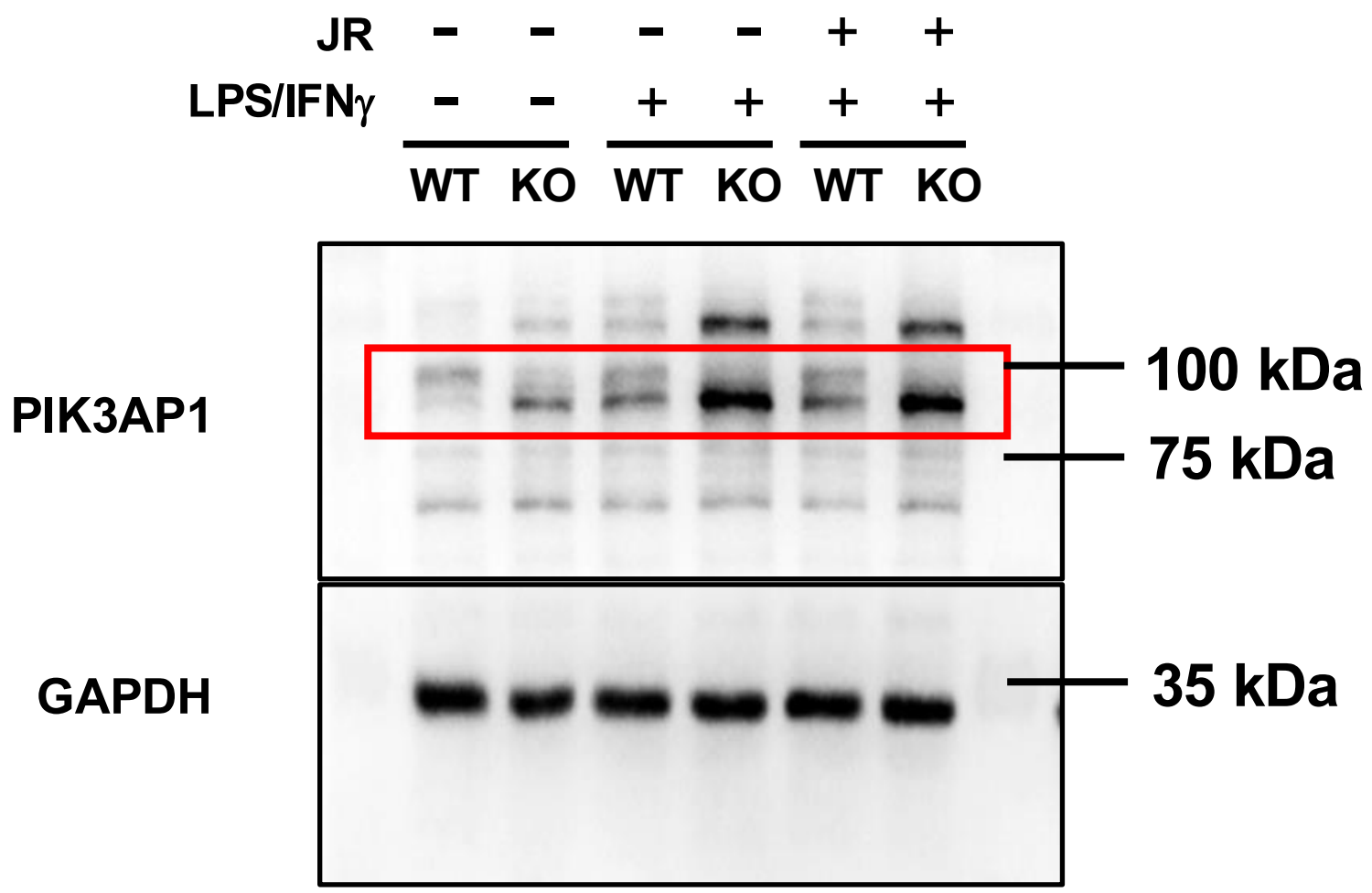

Figure 6

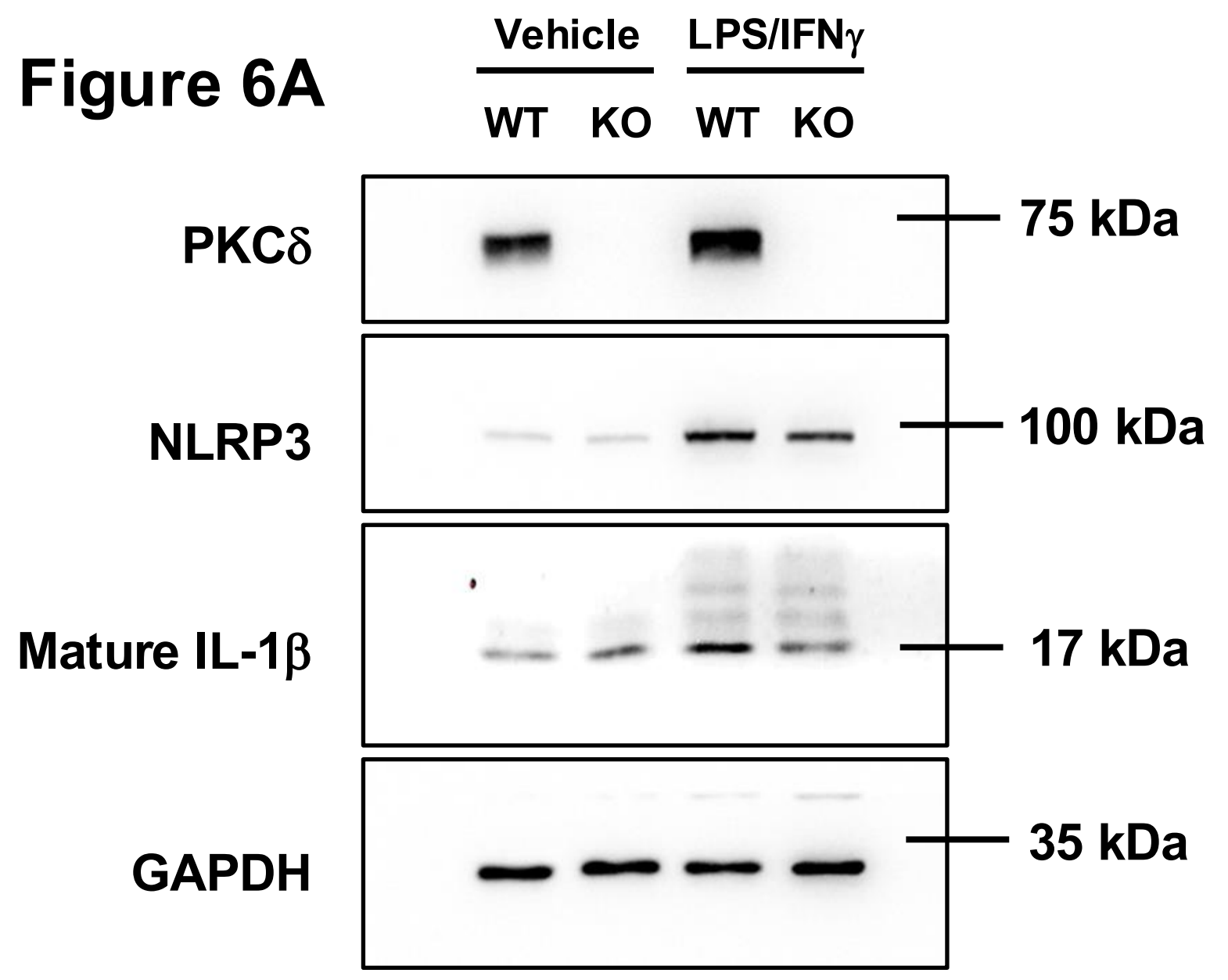
Figure 6A
Vehicle
LPS/IFNγ
WT
KO
WT
KO
PKCδ
75 kDa
NLRP3
100 kDa
Mature IL-1β
17 kDa
GAPDH
35 kDa

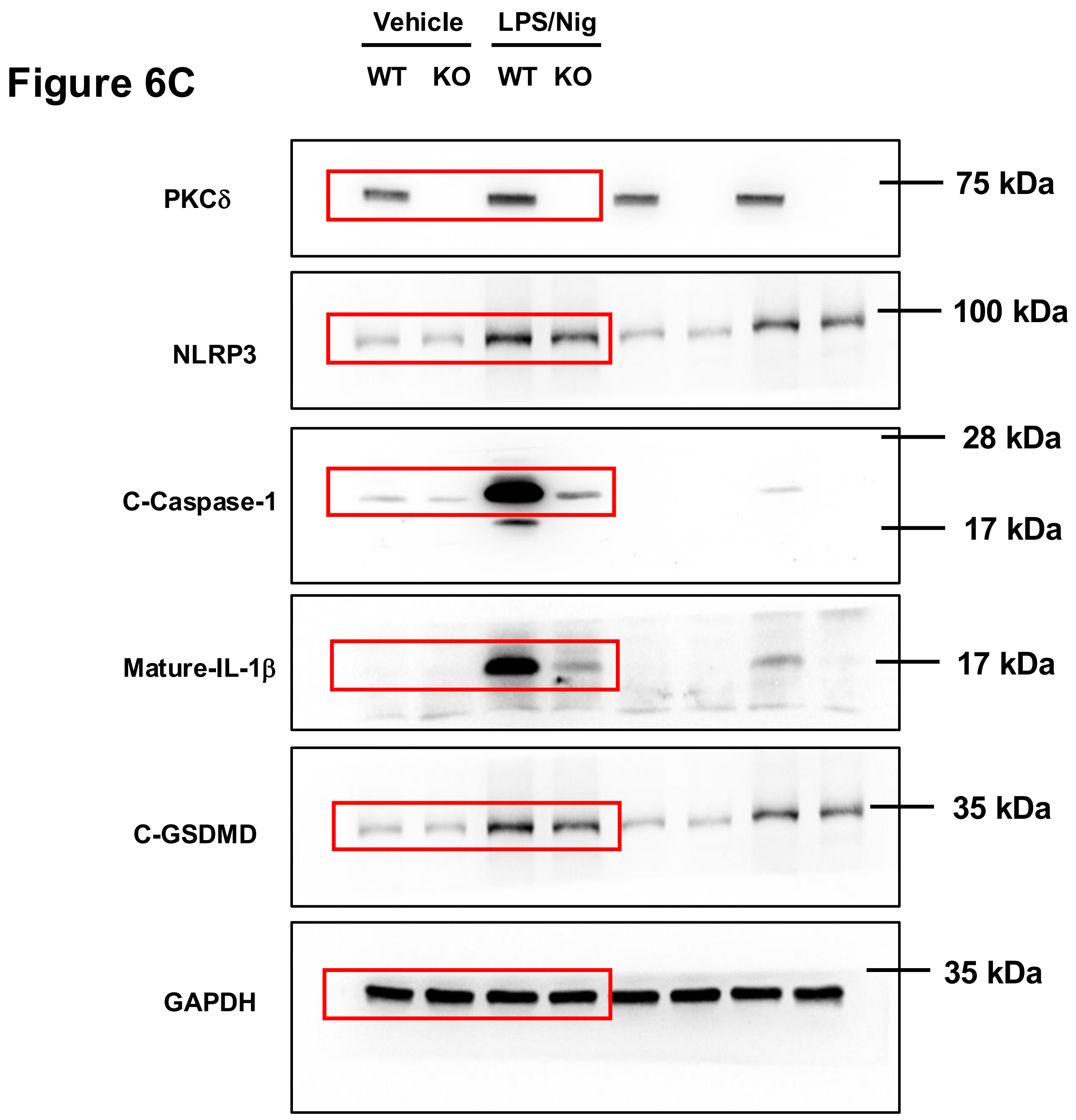
Figure 6C
Vehicle
LPS/Nig
WT
KO
WT
KO
PKCδ
75 kDa
NLRP3
100 kDa
C-Caspase-1
28 kDa
17 kDa
Mature-IL-1β
17 kDa
C-GSDMD
35 kDa
GAPDH
35 kDa

Figure 6

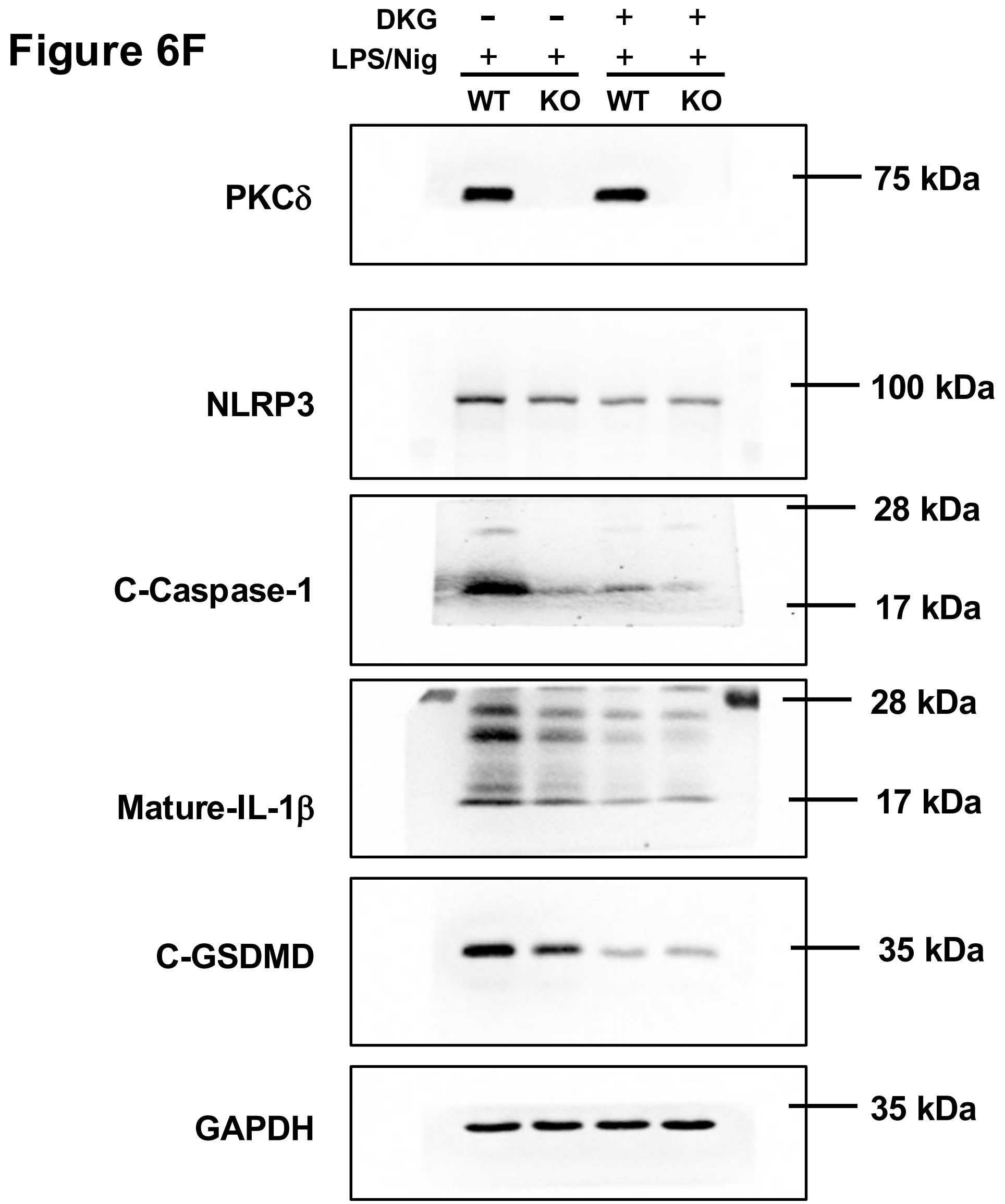

Figure 6F
DKG - - + +
LPS/Nig + + + +
WT KO WT KO
PKCδ
75 kDa
NLRP3
100 kDa
C-Caspase-1
28 kDa
17 kDa
Mature-IL-1β
28 kDa
17 kDa
C-GSDMD
35 kDa
GAPDH
35 kDa

Figure S4

Figure S4A

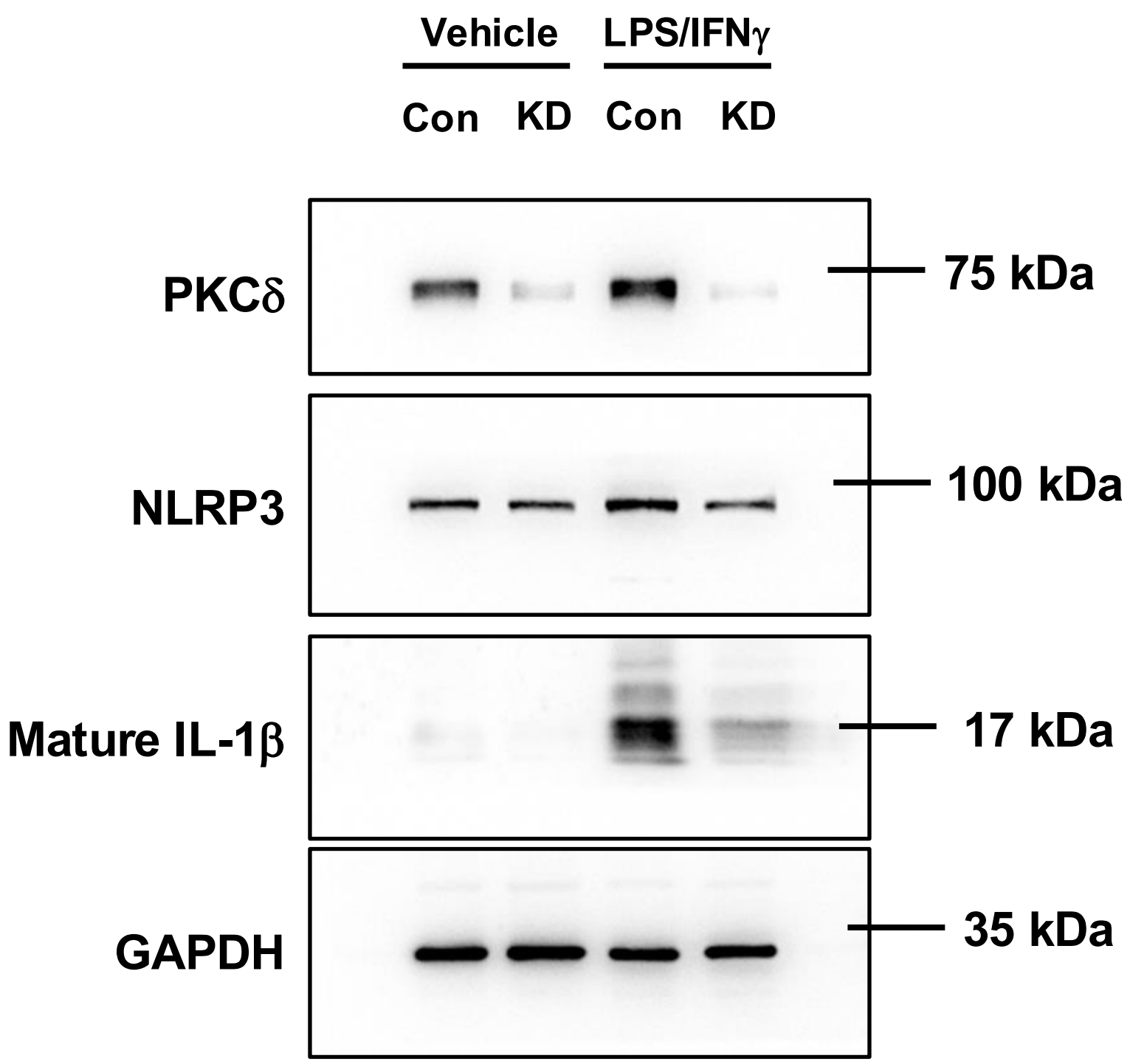

Figure S4C

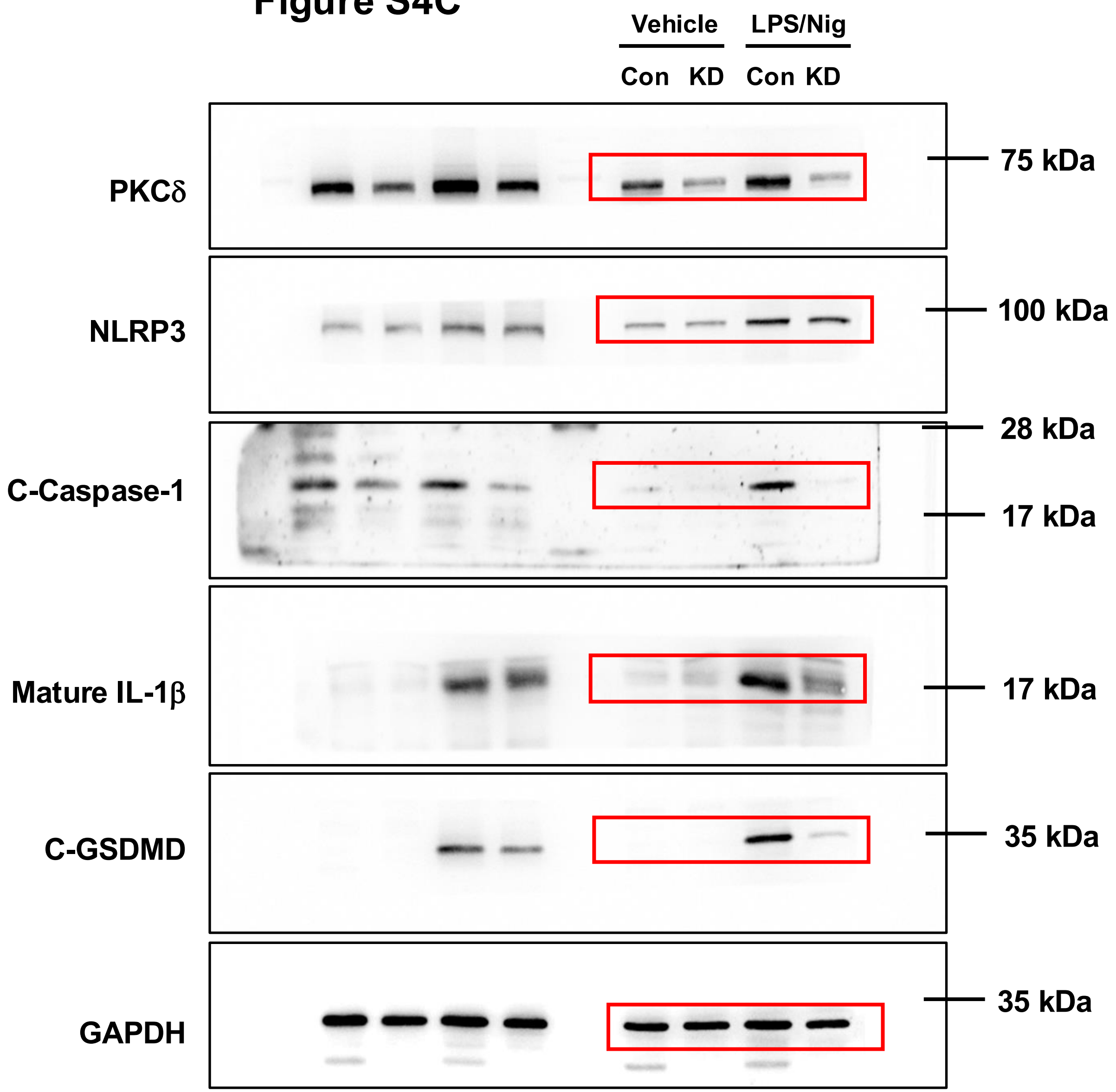

Figure S5

Figure S5C

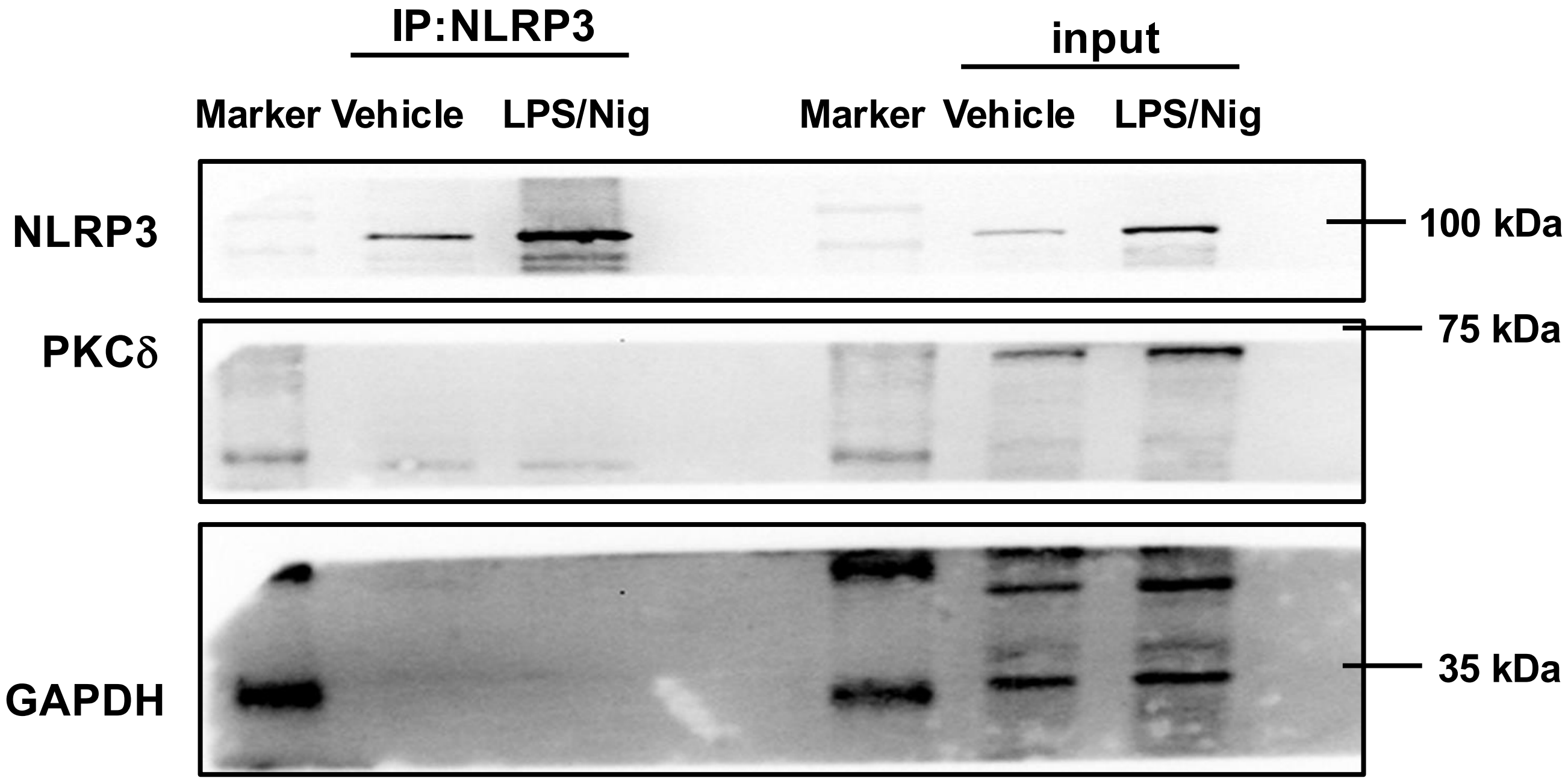

Supplement: Supplementary file 2 [file 12276_2026_1842_MOESM2_ESM.pdf]
